# Type I interferon signature and cycling lymphocytes in macrophage activation syndrome

**DOI:** 10.1172/JCI165616

**Published:** 2023-11-15

**Authors:** Zhengping Huang, Kailey E. Brodeur, Liang Chen, Du, Holly Wobma, Evan E. Hsu, Meng Liu, Joyce C. Chang, Margaret H. Chang, Janet Chou, Megan Day-Lewis, Fatma Dedeoglu, Olha Halyabar, James A. Lederer, Tianwang Li, Mindy S. Lo, Meiping Lu, Esra Meidan, Jane W. Newburger, Adrienne G. Randolph, Mary Beth Son, Robert P. Sundel, Maria L. Taylor, Huaxiang Wu, Qing Zhou, Scott W. Canna, Kevin Wei, Lauren A. Henderson, Peter A. Nigrovic, Pui Y. Lee

**Affiliations:** 1 Division of Immunology, Boston Children’s Hospital, Harvard Medical School, Boston, Massachusetts, USA.; 2 Department of Rheumatology and Immunology, Guangdong Second Provincial General Hospital, Southern Medical University, Guangzhou, China.; 3 Department of Rheumatology, The Second Affiliated Hospital of Zhejiang University School of Medicine, Hangzhou, China.; 4 Center for Data Sciences, Brigham and Women’s Hospital and Harvard Medical School, Boston, Massachusetts, USA.; 5 Department of Rheumatology, Immunology and Allergy, Zhejiang University School of Medicine, Hangzhou, China.; 6 Department of Cardiology, Department of Pediatrics, and; 7 Department of Anesthesiology, Critical Care, and Pain Medicine, Boston Children’s Hospital, Harvard Medical School, Boston, Massachusetts, USA.; 8 The MOE Key Laboratory of Biosystems Homeostasis and Protection, Life Sciences Institute, Zhejiang University, Hangzhou, China.; 9 Division of Rheumatology, Children’s Hospital of Philadelphia, Philadelphia, Pennsylvania, USA.; 10 Division of Rheumatology, Inflammation, and Immunity, Brigham and Women’s Hospital, Harvard Medical School, Boston, Massachusetts, USA.

**Keywords:** Immunology, Inflammation, Cytokines

## Abstract

**BACKGROUND:**

Macrophage activation syndrome (MAS) is a life-threatening complication of Still’s disease (SD) characterized by overt immune cell activation and cytokine storm. We aimed to further understand the immunologic landscape of SD and MAS.

**METHOD:**

We profiled PBMCs from people in a healthy control group and patients with SD with or without MAS using bulk RNA-Seq and single-cell RNA-Seq (scRNA-Seq). We validated and expanded the findings by mass cytometry, flow cytometry, and in vitro studies.

**RESULTS:**

Bulk RNA-Seq of PBMCs from patients with SD-associated MAS revealed strong expression of genes associated with type I interferon (IFN-I) signaling and cell proliferation, in addition to the expected IFN-γ signal, compared with people in the healthy control group and patients with SD without MAS. scRNA-Seq analysis of more than 65,000 total PBMCs confirmed IFN-I and IFN-γ signatures and localized the cell proliferation signature to cycling CD38^+^HLA-DR^+^ cells within CD4^+^ T cell, CD8^+^ T cell, and NK cell populations. CD38^+^HLA-DR^+^ lymphocytes exhibited prominent IFN-γ production, glycolysis, and mTOR signaling. Cell-cell interaction modeling suggested a network linking CD38^+^HLA-DR^+^ lymphocytes with monocytes through IFN-γ signaling. Notably, the expansion of CD38^+^HLA-DR^+^ lymphocytes in MAS was greater than in other systemic inflammatory conditions in children. In vitro stimulation of PBMCs demonstrated that IFN-I and IL-15 — both elevated in MAS patients — synergistically augmented the generation of CD38^+^HLA-DR^+^ lymphocytes, while Janus kinase inhibition mitigated this response.

**CONCLUSION:**

MAS associated with SD is characterized by overproduction of IFN-I, which may act in synergy with IL-15 to generate CD38^+^HLA-DR^+^ cycling lymphocytes that produce IFN-γ.

## Introduction

Still’s disease (SD) is characterized by recurrent fever, skin rash, arthritis, and systemic inflammation. SD was first recognized in children and is commonly called systemic juvenile idiopathic arthritis (sJIA) when the age of onset is under 16 years (1, 2). The disease also occurs in adults, where it is often termed adult-onset SD (AOSD) (3). In both children and adults, SD is a severe inflammatory condition with greater morbidity and mortality than other forms of arthritis (4, 5).

A major cause of fatality in SD is macrophage activation syndrome (MAS), a cytokine storm syndrome caused by hyperactivation of immune cells that occurs in 10%–30% of patients with SD (6, 7). The biology of MAS closely resembles that of familial hemophagocytic lymphohistiocytosis (HLH), caused by genetic variants that disrupt the lytic function of NK cells and cytotoxic T lymphocytes (8, 9). The inability to remove activated T lymphocytes perpetuates a vicious cycle of macrophage activation and dysregulated cytokine production. The overwhelming inflammatory response from continuous immune activation can quickly lead to multi-organ system failure and death.

Clinically, MAS is characterized by fever, pancytopenia, coagulopathy, hepatosplenomegaly, hyperferritinemia, and cytokine storm (10, 11). Rapid diagnosis and aggressive treatment are needed to prevent mortality. Whereas IL-1 and IL-6 are established therapeutic targets for SD (12, 13), IL-18 and IFN-γ (also known as type II IFN) are key contributors to the pathophysiology of MAS, and high levels of these cytokines as well as their downstream products can serve as diagnostic markers of MAS (14–17). A monoclonal antibody against IFN-γ was recently approved for the treatment of familial HLH and has also been shown to be effective in MAS associated with SD (18, 19).

The immunologic link between SD and MAS is not well defined. Multiple studies have examined gene expression signatures in SD, but the transcriptomic landscape of MAS has not been examined in detail. In this study, we performed high-dimensional analysis of SD and MAS by bulk RNA-Seq, single-cell RNA-Seq (scRNA-Seq), and mass cytometry. Here, we describe the immunologic landscape of MAS including a type I IFN (IFN-I) signature and an expansion of cycling T lymphocyte and NK cell subsets.

## Results

### Activation of IFN and cell cycling pathways in MAS.

To understand the gene expression profile across the disease spectrum of SD, we performed bulk RNA-Seq analysis of PBMC from patients with active SD without MAS (SD group; *n* = 10), SD patients with MAS (MAS group; *n* = 10), and people in a healthy control group (*n* = 18; 10 children and 8 adults). Patient demographics, laboratory features, and medication usage at the time of sampling are described in Supplemental Table 1 (supplemental material available online with this article; https://doi.org/10.1172/JCI165616DS1). Among patients with MAS, 6 were newly diagnosed with SD and samples were obtained before initiation of immunosuppressive therapy. The remainder were patients with a known history of SD and were receiving anakinra, corticosteroids, and/or tofacitinib at the time of sample collection. In the SD group, all patients had active disease as determined by the treating provider on the day of sample collection. Consistent with previous studies, SD was associated with marked elevation of plasma IL-18 levels (median 17,220 pg/mL), while the MAS group showed even higher IL-18 levels (median 101,546 pg/mL) as well as increased IFN-γ production, as reflected by high levels of the IFN-γ–inducible chemokine CXCL9 (median 3,889 pg/mL versus 366 pg/mL in the SD group; Supplemental Table 1) (14, 15, 20).

**Figure 1:**
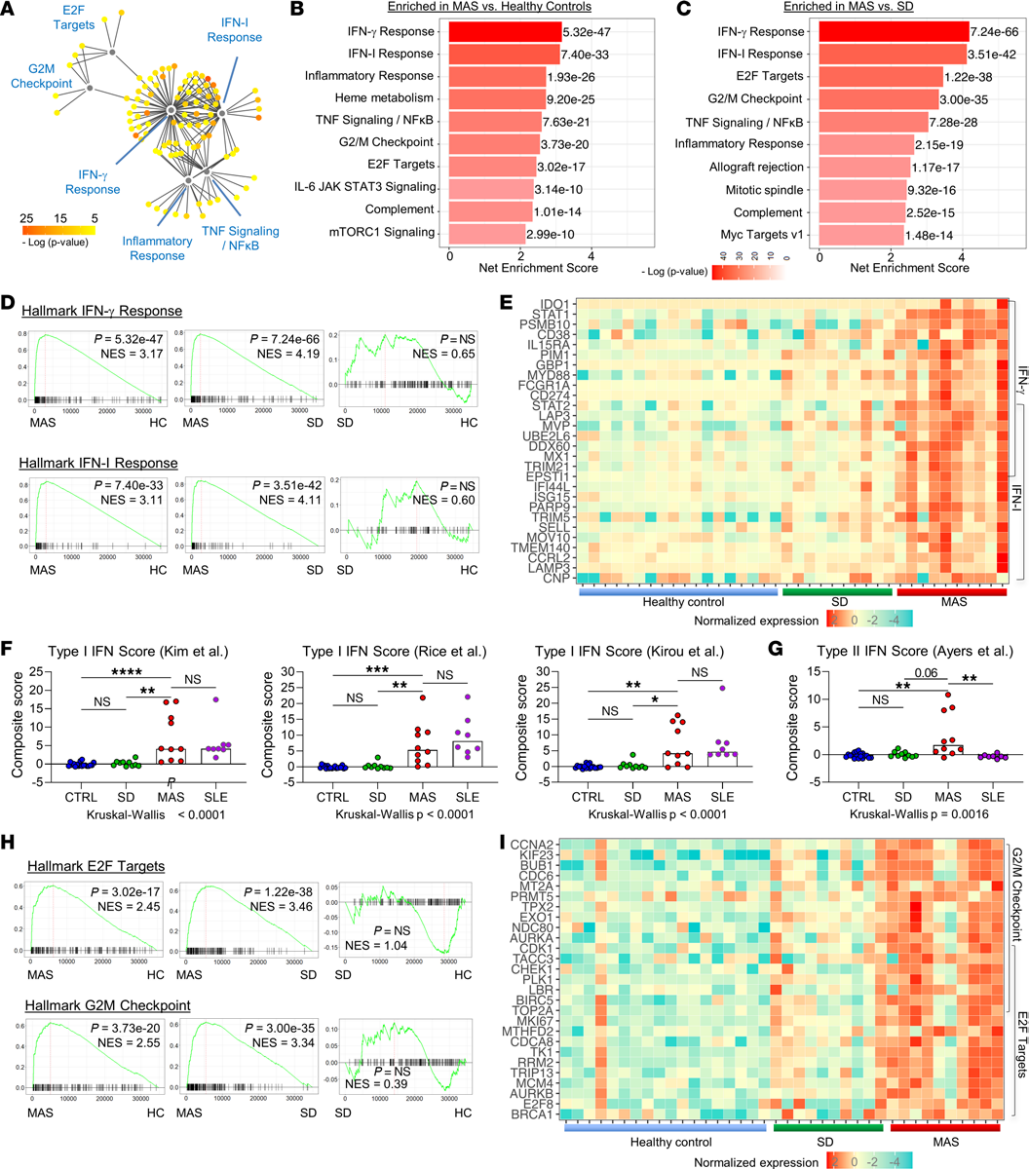
Presence of IFN-I and IFN-γ signatures in patients with MAS. (**A**) Cytoscape plot of top upregulated genes in patients with MAS (*n* = 10) compared with people in the healthy control group (HC; *n* = 18). Genes were grouped by hallmark gene sets. (**B**) GSEA of patients with MAS versus people in the healthy control group (**C**) patients with MAS versus patients with active sJIA without MAS (*n* = 10). Net enrichment score and *P* value of the top 10 enriched gene sets for each comparison are displayed. (**D**) GSEA plots of hallmark IFN-I response and IFN-γ response gene sets, and (**E**) heatmap display of top leading-edge genes in each pathway comparing patients with MAS, active sJIA without MAS, and people in the healthy control group. Genes included in both IFN-I response and IFN-γ response gene sets are indicated by overlapping brackets. (**F**) Comparison of composite gene set score derived from published IFN-I signature and (**G**) IFN-γ signature gene sets. Patients with active SLE (*n* = 8) were included for comparison. (**H**) GSEA plots of hallmark E2F targets and G2M checkpoint gene sets and (**I**) heatmap display of leading-edge genes in each pathway comparing patients with MAS, patients with active sJIA without MAS, and people in the healthy control group. Genes included in both E2F targets and G2M checkpoint pathways are indicated by overlapping brackets. Statistical analysis: bars in panels **F** and **G** represent the median. Kruskal-Wallis test was used for comparison of multiple groups and Dunn’s correction was applied for the indicated comparisons. *P < 0.05, **P < 0.01, ***P < 0.001, ***P < 0.0001.

Network analysis of upregulated genes in the MAS group compared with healthy controls showed clustering of genes associated with IFN-I signaling, IFN-γ signaling, and inflammatory response (Figure 1A and Supplemental Figure 1A). Consistent with elevated levels of IFN-γ in MAS (14), IFN-γ response genes were highly enriched in the MAS group compared with the healthy control group based on gene set enrichment analysis (GSEA) using the validated hallmark gene set collection (Figure 1B) (21, 22). Patients with MAS also displayed a strong enrichment of genes that reflect IFN-I signaling. These patterns were specific to MAS, as the enrichment of IFN-I and IFN-γ signaling was also observed when compared with the SD group but not enriched significantly in the SD group compared with the healthy control group (Figure 1, C and D). Heatmap display of genes in the leading edge illustrated that genes associated with IFN-I signaling and IFN-γ signaling, including some that are regulated by both pathways, were upregulated among most patients with MAS but rare in those with uncomplicated SD (Figure 1E).

Unlike IFN-γ, a role for IFN-I in MAS has not been appreciated. Given the overlap in genes regulated by IFN-I and IFN-γ, we employed additional published gene sets to confirm the IFN-I signature. Using a validated method to calculate a standardized gene expression score from several established IFN-I gene sets (23–25), we found upregulation of IFN-I–inducible genes in patients with MAS comparable to the levels observed in patients with systemic lupus erythematosus (SLE), an autoimmune disease in which IFN-I dysregulation plays a pathogenic role (Figure 1F). In contrast, the upregulation of IFN-γ–inducible gene sets from published studies (26, 27) was more prominent in MAS than in SLE (Figure 1G and Supplemental Figure 1B). To further demonstrate the specificity of the IFN-I signature in MAS, we cocultured plasma from patients and people in the healthy control group with 293T IFN-I luciferase reporter cells that respond minimally to IFN-γ (Supplemental Figure 1C). Plasma from patients with MAS induced significantly higher levels of luciferase activity compared with plasma from healthy controls and uncomplicated SD (Supplemental Figure 1D).

Also among the top biological pathways enriched in MAS compared with both the healthy control and SD groups were E2F targets and G2/mitosis (G2/M) checkpoint gene sets, both of which reflect cell cycling and proliferation (Figure 1, B, C, and H). A heatmap display of leading-edge genes showed prominent expression of these gene sets in most patients with MAS (Figure 1I). Taken together, bulk RNA-Seq of PBMCs revealed gene signatures of IFN-I signaling, IFN-γ signaling, and cell cycling/proliferation in MAS.

### Immunologic landscape of SD and MAS at single-cell resolution.

**Figure 2:**
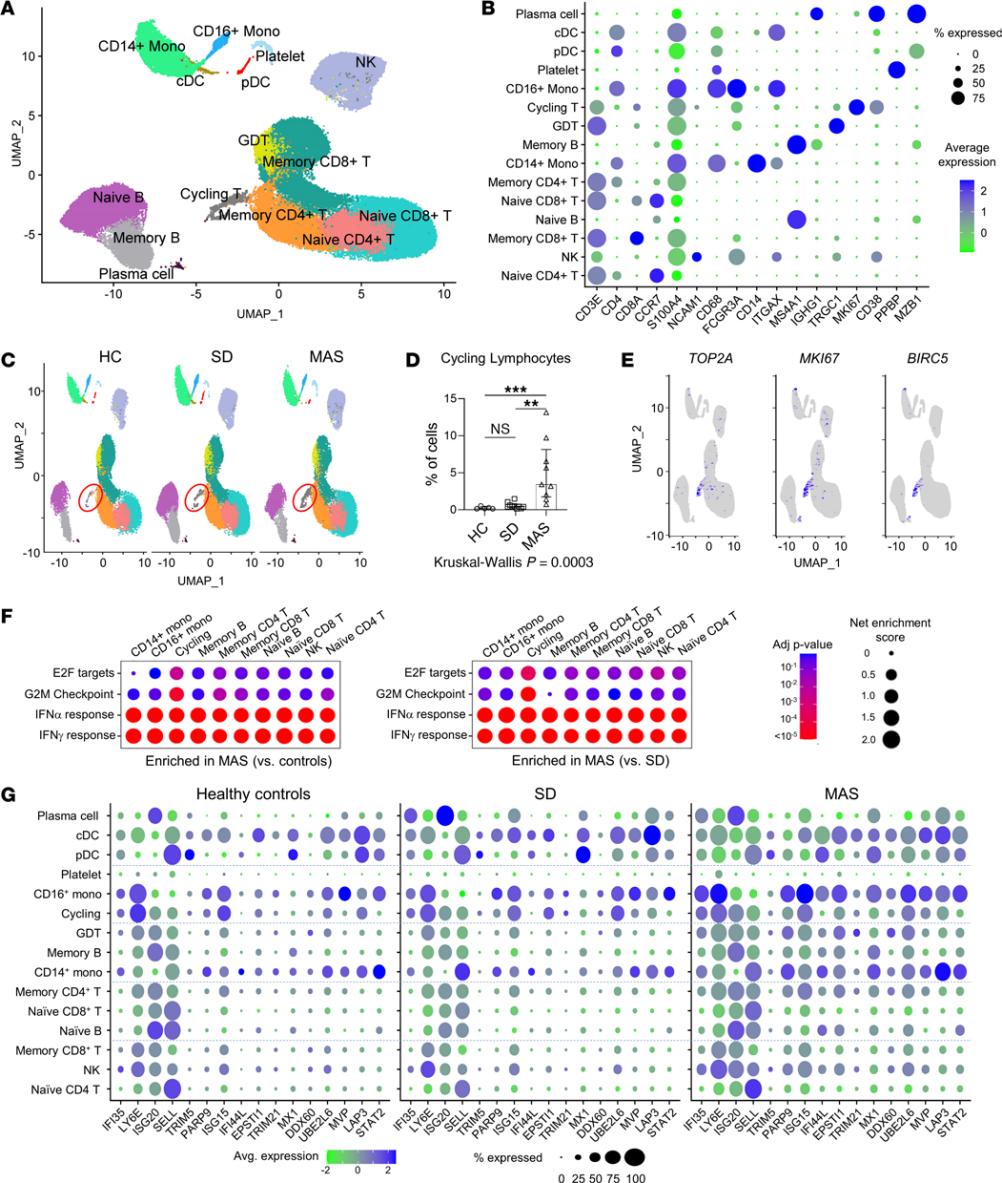
Single cell transcriptomic landscape of SD and MAS. (**A**) UMAP display of PBMC (65,131 cells) concatenated from people in the healthy control group (*n* = 5), patients with SD without MAS (*n* = 10) and patients with MAS (*n* = 9). Cell subsets are labeled based on the expression of lineage-defining genes. (**B**) Cluster plot of lineage-defining markers for major leukocyte subsets. Size of circles represents percentage of cells expressing the indicated marker and color indicates strength of expression. (**C**) UMAP display of PBMC in people in the healthy control group, and patients with active SD with or without MAS. Cell populations are as defined in panel **A**. Red circle indicates cycling lymphocytes. (**D**) Quantification of cycling lymphocytes in people in the healthy control group and patients with SD with or without MAS. (**E**) Feature plot illustrating the expression of cell proliferation markers TOP2A, MKI67, and BIRC5. (**F**) Cluster plot of GSEA comparing hallmark gene sets (IFN-I response, IFN-γ response, E2F targets, and G2M checkpoint) in major immune cell populations. The MAS group was compared with the healthy control group (left) and patients with SD without MAS (right). Size of circles represents the degree of enrichment and color indicates *P* value. (**G**) Cluster plot of IFN-stimulated gene expression among leukocyte subsets. Size of circles represents percentage of cells expressing the indicated marker, while color indicates strength of expression. Statistical analysis: Bars represent the median and error bars indicate interquartile range in panel **D**. Kruskal-Wallis test was used for comparison of multiple groups and Dunn’s correction was applied for the indicated comparisons. **P < 0.01, ***P < 0.001.

To explore the cellular context of these gene signatures, we performed scRNA-Seq of PBMC from people in the healthy control group (*n* = 5), patients with SD without MAS (*n* = 10), and patients with MAS (*n* = 9). The number of cells analyzed for each individual was normalized to account for the cytopenia associated with MAS. A total of 65,130 cells that satisfied quality control metrics were included in the final analysis. Unsupervised clustering identified 15 major cell clusters as displayed by Uniform Manifold Approximation and Projection (UMAP), and these populations were then annotated based on defined markers (Figure 2, A and B) (28). Highly expressed genes specific to each population are displayed in Supplemental Figure 2A.

Quantitative comparison of cell populations revealed an expansion of a distinctive lymphocyte population in patients with MAS compared with the SD and healthy control groups (Figure 2, C and D and Supplemental Figure 2B). These cells uniquely expressed markers of proliferation, including *MKI67*, *TOP2A,* and *BIRC5* (Figure 2E) and so are termed “cycling lymphocytes” in a recent scRNA-Seq study on patients with COVID-19 (28). GSEA of cell subsets showed that the cycling lymphocytes in the MAS group were largely responsible for the enrichment of E2F targets and G2/M checkpoint gene sets observed in the bulk RNA-Seq studies (Figure 2F). In contrast, the enrichment of IFN-I and IFN-γ response gene sets was observed across most immune cell subsets in MAS. Display of IFN-stimulated genes confirmed the general increase in expression levels in the MAS group, most prominently among monocytes and dendritic cells (Figure 2G).

### Profiling of cycling lymphocytes associated with MAS.

**Figure 3:**
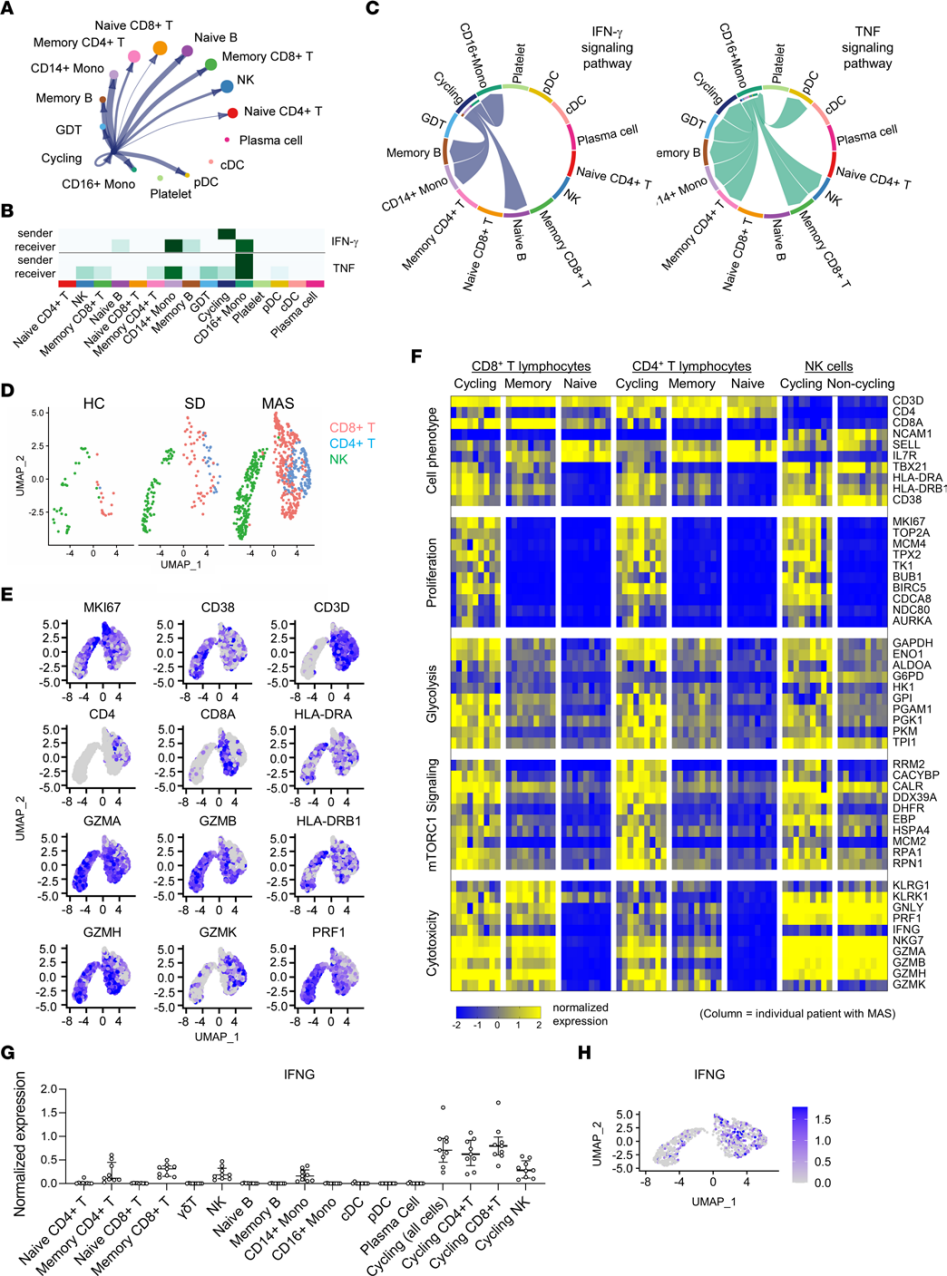
scRNA-Seq analysis of cycling lymphocyte subsets in patients with MAS. (**A**) Circular plot illustrating cellular communication between cycling lymphocytes and other PBMC subsets. Cell populations are as defined in Figure 2. (**B**) heatmap display and (**C**) chord diagram of projected sender(s) and receiver(s) of IFN-γ and TNF signaling in PBMC cell subsets based on CellChat analysis of scRNA-Seq data from patients with MAS. (**D**) UMAP display of cycling lymphocytes from people in the healthy control group (*n* = 5), patients with SD without MAS (*n* = 10), and patients with MAS (*n* = 9). (**E**) Feature plot illustrating the expression of lineage and phenotypic markers in cells from patients with MAS. Cell populations are as defined in panel **D**. (**F**) Heatmap display of gene expression in cycling lymphocytes and major T and NK cell subsets in patients with MAS. Each column represents data from a single patient. Genes were selected from the leading edge of GSEA comparing cycling lymphocytes and all other cell subsets. (**G**) Quantification of average IFNG expression in indicated immune cell subsets in patients with MAS. Cycling lymphocytes were shown as a group and as individual subsets of T cells and NK cells. (**H**) Feature plot illustrating expression of *IFNG* in cycling lymphocytes from patients with MAS.

Next, we explored cell-cell interactions among immune cells in people in the healthy control group, patients with SD without MAS, and patients with MAS based on the scRNA-Seq data (29). CellChat analysis identified outgoing and incoming signaling pathways in the major cell subsets in all 3 groups (Supplemental Figure 3, A–C). Among the cell communication pathways specific to the MAS group were IFN-γ signaling and cell death pathways (Fas ligand, CD30 and LIGHT/TNFSF14) mediated by T lymphocytes (Supplemental Figure 3, A and D). The cycling lymphocytes in the MAS group were predicted to interact with numerous other cell subsets (Figure 3A). Cycling lymphocytes were projected to be primary producers (sender) of IFN-γ, which stimulates CD14^+^ and CD16^+^ monocytes (receivers; Figure 3, B and C). On the other hand, CD16^+^ monocytes were predicted to be the main source of TNF, which acts on cycling lymphocytes, monocytes, memory T cells, and NK cells (Figure 3C).

Focused profiling revealed that cycling lymphocytes enriched in MAS are comprised of CD4^+^ T cells, CD8^+^ T cells, and NK cells (Figure 3D and Supplemental Figure 4, A and B). All 3 subsets expressed the activation markers *CD38* and *HLA-DR* (Figure 3E). Compared with naive and memory T lymphocyte subsets and NK cells from patients with MAS, the cycling subpopulations displayed enhanced expression of genes involved in cell proliferation, glycolysis, and mechanistic target of rapamycin (mTOR) signaling (Figure 3F), suggesting that these cells are highly metabolically active. Cycling CD8^+^ T cells and NK cells exhibited prominent expression of perforin and granzymes, suggesting cytolytic function. CD4^+^ and CD8^+^ cycling T cells showed higher levels of *IFNG* transcript than all other cell types studied (Figure 3, G and H), supporting the earlier cell-cell interaction data suggesting that these cells are primary producers of IFN-γ in MAS (Figure 3B).

We performed mass cytometry analysis on a subset of PBMC samples in parallel using a broad immunophenotyping panel (healthy control group, *n* = 5; SD group, *n* = 7; MAS group, *n* = 5). UMAP analysis defined the major leukocyte subsets across all samples (Supplemental Figure 5A). Profiling of CD4^+^ T cells, CD8^+^ T cells, and NK cells identified a subset of cells with prominent surface expression CD38 and HLA-DR mostly seen in patients with MAS (Supplemental Figure 5, B–E). CD38^+^HLA-DR^+^ cells also expressed the proliferation marker Ki-67. Therefore, surface protein staining of CD38 and HLA-DR marked the cycling lymphocyte subsets identified by scRNA-Seq. Taken together, our multiomic analysis by bulk RNA-Seq, scRNA-Seq, and mass cytometry collectively demonstrated an expansion of cycling lymphocytes in patients with MAS.

### CD38^+^ HLA-DR^+^ lymphocytes in MAS and other pediatric inflammatory diseases.

**Figure 4:**
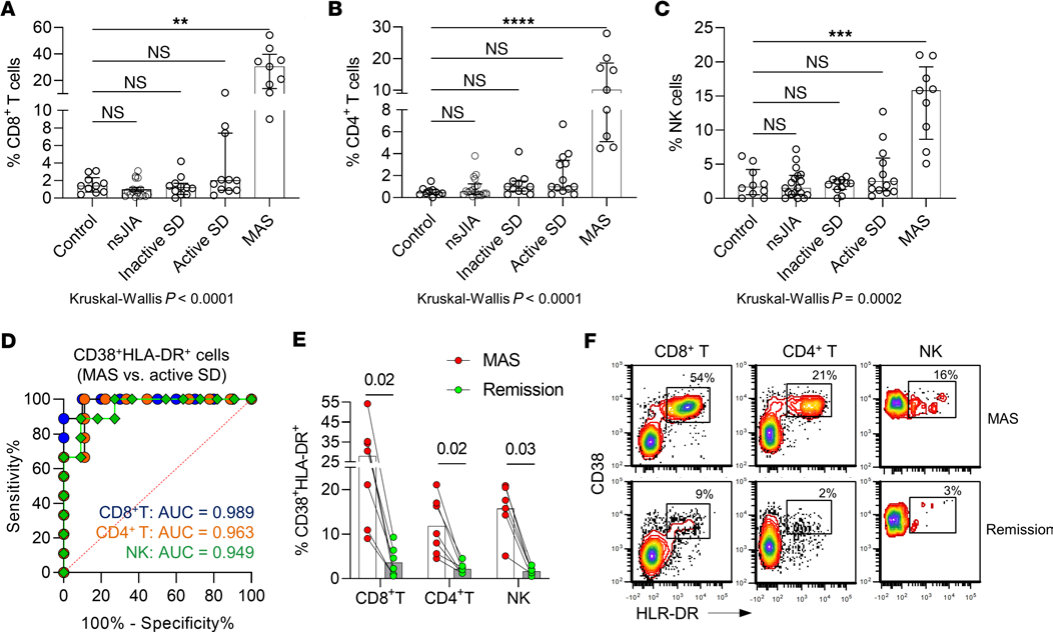
Flow cytometry analysis of CD38^+^HLA-DR^+^ lymphocyte subsets. (**A**) Quantification of CD38^+^HLA-DR^+^CD8^+^ T cells, (**B**) CD38^+^HLA-DR^+^CD4^+^ T cells, and (**C**) CD38^+^HLA-DR^+^C56^+^ NK cells in healthy children (*n* = 10), and patients with nonsystemic JIA (*n* = 15), inactive SD (*n* = 11), active SD without MAS (*n* = 11), or active SD with MAS (*n* = 9). (**D**) Receiver operator characteristic curve illustrating the utility of using CD38^+^HLA-DR^+^ lymphocytes (as a percentage of T cell or NK cell pool) in distinguishing cases of SD-associated MAS (*n* = 9) from patients with active SD without MAS (*n* = 11). (**E**) Quantification and (**F**) representative flow cytometry plot of CD38^+^HLA-DR^+^ lymphocyte subsets in 6 patients during MAS and after resolution of MAS. Statistical analysis: Bars in panels **A**–**C** represent the median and error bars indicate interquartile range. Kruskal-Wallis test was used for comparison of multiple groups and Dunn’s correction was applied for the comparisons with the control group (panels **A**–**C**). Wilcoxon signed-rank test was used for panel **E**. *P < 0.05, **P < 0.01, ***P < 0.001, ***P < 0.0001.

To investigate the clinical correlates of CD38^+^ HLA-DR^+^ T cells and NK cells, we performed flow cytometry analysis on PBMCs from people in the healthy control group (*n* = 10), patients with SD stratified by disease state at the time of sampling (inactive SD, *n* = 11; active SD without MAS, *n* = 11; MAS, *n* = 9), and children with other types of JIA (nonsystemic JIA; *n* = 15). The study groups are described in Supplemental Table 2. Supporting the data from mass cytometry and scRNA-Seq, patients with MAS displayed markedly higher proportions of CD38^+^ HLA-DR^+^ cells within CD4^+^ T cell, CD8^+^ T cell, and NK cell populations (Figure 4, A–C). Aside from a slight increase in CD38^+^ HLA-DR^+^ CD4^+^ T cells in patients with active SD versus people in the healthy control group, the proportions of these T cell and NK cell subsets were comparable in the control group and patients with nonsystemic JIA, inactive SD, or active SD without MAS. Receiver operator characteristic curves revealed that the proportions of CD38^+^ HLA-DR^+^ T cells and NK cells were highly sensitive and specific in discriminating MAS from uncomplicated active SD (Figure 4D). This association with MAS was further supported by follow-up samples from 6 patients that showed significant reductions in CD38^+^ HLA-DR^+^ T cells and NK cells after the resolution of MAS (Figure 4, E and F).

CD8^+^ T cells that express CD38 and HLA-DR have been described in viral infections and more recently in MAS/HLH as well as in multisystem inflammatory syndrome in children (MIS-C) associated with COVID-19 (30–33), although the reason for their expansion in these condition is unknown. To investigate how the expansion of CD38^+^ HLA-DR^+^ T cells and NK cells in SD-associated MAS compares with other inflammatory diseases, we examined patients with Kawasaki disease (*n* = 10), acute viral infection (*n* = 18), MIS-C (*n* = 20), juvenile dermatomyositis (JDM) (*n* = 11), SLE (*n* = 9), and MAS secondary to other causes (*n* = 5). Details of these study groups are provided in Supplemental Table 2.

**Figure 5:**
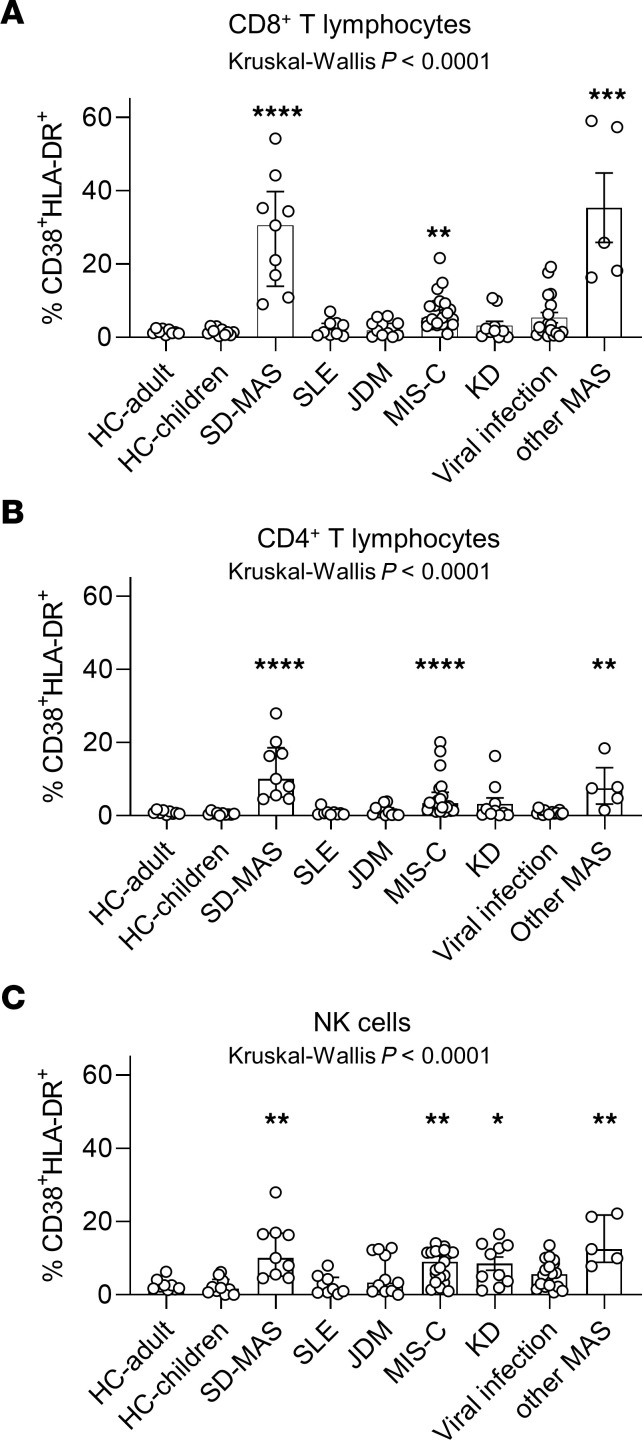
Quantification of CD38^+^HLA-DR^+^ lymphocytes in pediatric inflammatory conditions. (**A**) Quantification of CD38^+^HLA-DR^+^ CD8^+^ T cells, (**B**) CD38^+^HLA-DR^+^ CD4^+^ T cells, and (**C**) CD38^+^HLA-DR^+^ C56^+^ NK cells in healthy children (*n* = 11), healthy adults (*n* = 10), and patients with SD-associated MAS (n =9; as displayed in Figure 4), MIS-C associated with COVID-19 (*n* = 20), Kawasaki disease (*n* = 10), viral infections (*n* = 18), JDM (*n* = 11), and SLE (*n* = 9), or MAS secondary to other causes (*n* = 6). Statistical analysis: Bars represent median and error bars indicate interquartile range. Kruskal-Wallis test was used for comparison of multiple groups and Dunn’s correction was applied for the comparisons with the control group. *P < 0.05, **P < 0.01, ***P < 0.001, ***P < 0.0001.

Compared with people in the healthy control group, significant increases in CD38^+^ HLA-DR^+^ CD8^+^ cells and CD4^+^T cells were found in patients with MIS-C and MAS due to other causes (Figure 5, A and B). The degree of expansion in MAS secondary to other causes was comparable to MAS associated with SD. The levels in patients with MIS-C were lower than those in the MAS groups. Common viral infections in children (i.e. adenovirus, SARS-CoV-2, and parainfluenza virus) were associated with a slight increase in CD38^+^ HLA-DR^+^ T cells compared with children in the healthy control group (*P* = 0.04 for CD4^+^T cells and *P* = 0.10 for CD8^+^T cells). The expansion of CD38^+^ HLA-DR^+^ NK cells was more common among inflammatory conditions, with comparable levels in patients with MIS-C, Kawasaki Disease, viral infections, and MAS secondary to SD as well as MAS secondary to other causes (Figure 5C). In contrast, the expansion of CD38^+^ HLA-DR^+^ T cells and NK cells was not a characteristic of autoimmune diseases, including SLE, JDM, and nonsystemic JIA (Figure 4, A–C and Figure 5, A–C).

### In vitro generation of CD38^+^HLA-DR^+^ T cells.

We next explored the ontogeny of CD38^+^ HLA-DR^+^ cycling T cells associated with MAS. Since cytokine storm is central to the pathophysiology of MAS, we asked whether cytokines could induce the CD38^+^ HLA-DR^+^ phenotype in isolated T lymphocytes from healthy donors. We found that stimulation with IFN-α and IL-15 individually induced a small population CD38^+^HLA-DR^+^ cells within the CD8^+^ T cell pool after 48 hours (Supplemental Figure 6, A and B). This observation was not seen in cells cocultured with IL-1β, IL-4, IL-6, IL-10, IL-18, IL-27, TNF, or IFN-γ, even at the highest concentration of 100 ng/mL.

**Figure 6:**
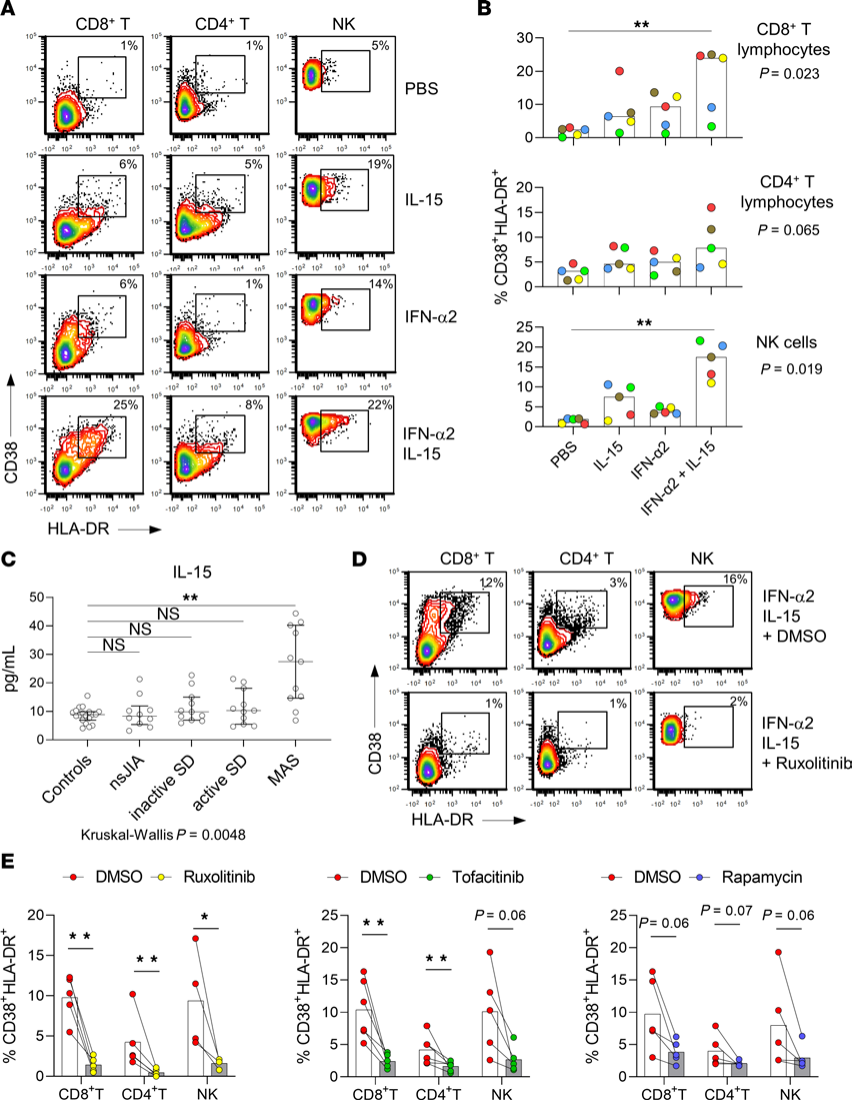
Generation of CD38^+^HLA-DR^+^ T lymphocytes and NK cells in vitro. (**A**) Representative flow cytometry plot and (**B**) quantification of CD38^+^HLA-DR^+^ lymphocyte subsets induced by coculturing PBMC from healthy controls (*n* = 5) with the indicated cytokine combinations for 2 days. Each color in panel **B** represents a unique healthy donor. Results for each healthy donor represent the average of duplicate samples. (**C**) Plasma IL-15 levels in healthy controls (*n* = 19), and patients with nonsystemic JIA (*n* = 10), inactive SD (*n* = 11), active SD without MAS (*n* = 11), or active SD with MAS (*n* = 11) as measured by proximity extension assay. (**D**) Representative flow cytometry plots and (**E**) Quantification of CD38^+^HLA-DR^+^ lymphocyte subsets induced by IL-15 (10 ng/mL) and IFN-α2 (10 ng/mL) in PBMCs (from 4–5 healthy donors) pretreated with ruxolitinib (100 nM), tofacitinib (100 nM), rapamycin (1 μM), or DMSO (vehicle control). Inhibitors or DMSO were added 30 minutes prior to IL-15 and IFN-α2 stimulation, and analysis was performed after 2 days. Statistical analysis: Bars represent median and error bars represent interquartile range. Kruskal-Wallis test was used for comparison of multiple groups in panels **B** and **C**, and Dunn’s correction was applied for the indicated comparisons. Mann-Whitney U test was applied for panel **E**. *P < 0.05, **P < 0.01.

Based on our earlier observation of IFN-I signature in MAS, we tested whether IFN-I induces the generation of CD38^+^HLA-DR^+^ T and NK cells in combination with other cytokines. Interestingly, the combination of IFN-α2 and IL-15 synergistically increased the expansion of CD38^+^ HLA-DR^+^ T cells and NK cells in PBMCs from healthy donors (Figure 6, A and B). Similar to the pattern seen in patients with MAS, the proportion of CD38^+^HLA-DR^+^ CD8^+^ T cells and NK cells induced by IFN-α2 and IL-15 was more prominent than CD4^+^ T cells. In contrast, the addition of IL-18 did not influence the differentiation of CD38^+^HLA-DR^+^ T cells and NK cells (Supplemental Figure 6C). Pretreatment with IL-18 for 24 hours also did not alter the effects of IFN-α2 and IL-15 (Supplemental Figure 6D).

IL-15 is produced by monocytes and dendritic cells and functions as a potent activator of T lymphocytes and NK cells (34). Supporting a potential role of IL-15 in MAS, plasma levels of IL-15 were significantly higher in patients with MAS compared with people in the healthy control, uncomplicated SD (inactive or active disease without MAS), and nonsystemic JIA groups (Figure 6C). We also measured plasma IL-15 levels in patients with active SLE (*n* = 10) and acute viral infection (*n* = 17). These conditions are associated with increased IFN-I production and both groups showed generally higher levels of IL-15 compared with the healthy control group (Supplemental Figure 6E). However, the degree of IL-15 elevation observed in these groups seemed milder than the MAS group. Of 11 samples, 6 in the MAS group displayed higher levels of IL-15 relative to all other groups (Supplemental Figure 6E). IL-15 levels were higher in the MAS group compared with patients with acute viral infection (*P* = 0.01). However, due to the small sample size and heterogeneity within the MAS group, likely related to treatment effects, the difference between the MAS and SLE groups was not statistically significant (*P* = 0.33).

The receptor for IL-15 comprises 3 components. When bound to the unique receptor IL-15RA (CD215), IL-15 activates target cells via the shared IL-2 receptor subunits IL-2RB (CD122) and IL-2RG (CD132) to induce downstream Janus kinase (JAK) phosphorylation. Flow cytometry analysis revealed that CD38^+^HLA-DR^+^ T cells (CD4^+^ and CD8^+^) from patients with MAS expressed low levels of IL-15RA but higher levels of IL-2RB and IL-2RG compared with their CD38^–^HLA-DR^–^ counterparts (Supplemental Figure 7A). IL-15 can be presented to target T lymphocytes and NK cells in cis (using IL-15RA expressed by the target cell), or in trans by binding to IL-15RA expressed by monocytes and dendritic cells (35, 36). We observed low but detectable expression of IL-15RA in major PBMC subsets from the healthy control group (Supplemental Figure 7B). We found that IFN-α2 and IL-15 can induce the generation of CD38^+^HLA-DR^+^ T cells from isolated T lymphocytes in vitro, without the addition of monocytes, dendritic cells, or other cell subsets (Supplemental Figure 7C), although with lower efficiency than PBMC.

Consistent with the cytotoxic profile predicted by our scRNA-Seq analysis, CD38^+^HLA-DR^+^CD8^+^ T cells generated by IFN-α2 and IL-15 in vitro expressed higher levels of perforin, granzyme A, and granzyme B than CD38^–^HLA-DR^–^CD8^+^ T cells (Supplemental Figure 8A). Upon activation by CD3/CD28 beads, CD38^+^HLA-DR^+^CD8^+^ T cells displayed increased degranulation, as measured by the externalization of CD107a (Supplemental Figure 8, B and C). A subset of CD38^+^HLA-DR^+^CD8^+^ T cells induced by IFN-α2 and IL-15, but not their CD38^–^HLA-DR^–^ counterparts, displayed evidence of cell proliferation based on Ki-67 staining (Supplemental Figure 8D). The extent of T cell proliferation in vitro induced by IFN-α2 and IL-15 was mild compared with T cell stimulation using anti-CD3/CD28 beads. Interestingly, the combination of IFN-α2 and IL-15 enhanced the proliferative effects induced by anti-CD3/CD28 beads (Supplemental Figure 8, D and E).

Finally, we analyzed potential therapeutic options to abrogate the generation of CD38^+^HLA-DR^+^ T and NK cells. JAK inhibitors are increasingly employed for the treatment of HLH and MAS due to the involvement of JAKs in the signaling of proinflammatory cytokines including IFN-γ (37). Consistent with the requirement of JAK1 downstream of IFN-I and IL-15 signaling, the JAK inhibitors ruxolitinib and tofacitinib effectively inhibited the development of CD38^+^ HLA-DR^+^ T and NK cells induced by these cytokines (Figure 6, D and E). Because the CD38^+^HLA-DR^+^ T and NK cells were highly proliferative and metabolically active with enhanced mTORC1 signaling according to our scRNA-Seq data, we tested the effect of the mTOR inhibitor rapamycin. Regulation of immunometabolism by mTORC1 governs effector T cell function, and overt activation of this pathway was shown to cause features of SD and MAS in a murine model (38). Rapamycin attenuated the expansion of CD38^+^HLA-DR^+^ T and NK cells induced by IFN-α2 and IL-15, although the degree of inhibition appeared mild compared with JAK inhibitors, and statistical significance was borderline (*P* = 0.06 to 0.07). These data support a role for IFN-I and IL-15 in promoting the differentiation of CD38^+^ HLA-DR^+^ T and NK cells and illustrate the potential utility of JAK inhibitors and possibly mTOR inhibitors to control the expansion of these cells in MAS.

## Discussion

MAS is a dangerous complication of SD with high mortality and morbidity that requires prompt recognition and treatment. In this study, we utilized high-dimensional approaches to improve our understanding of the immunologic landscape of SD and MAS. Bulk RNA-Seq, scRNA-Seq, and mass cytometry studies together established (a) the presence of both IFN-I and IFN-γ signatures in MAS; (b) a gene signature of cellular proliferation that localized to CD4^+^ and CD8^+^ T lymphocytes and NK cells marked by the expression of CD38 and HLA-DR, cells we term cycling lymphocytes; (c) greater expansion of CD38^+^HLA-DR^+^ T lymphocytes and NK cells in MAS compared with other pediatric inflammatory conditions, including MIS-C and viral infections; and (d) synergistic effects of IFN-I and IL-15 in driving the development of CD38^+^HLA-DR^+^ T lymphocytes in vitro.

### Cycling lymphocytes as producers of IFN-γ in MAS.

Ineffective clearance of activated cytotoxic lymphocytes leading to a vicious cycle of immune cell activation and cytokine production is considered the mechanistic basis of HLH and MAS (6, 8). While genetic defects that compromise immune synapse formation or lytic function of cytotoxic lymphocytes are responsible for familial HLH — and also serve as susceptibility factors in MAS — the etiology of MAS associated with SD is still poorly defined. The expansion of CD38^+^HLA-DR^+^CD8^+^ T cells appears to be a shared feature of HLH and MAS. Chaturvedi and colleagues found that more than 7% of CD38^+^HLA-DR^+^ cells within the CD8^+^ T cell pool distinguished patients with HLH from those with sepsis (30). De Matteis et al. recently showed similar findings in MAS associated with SD as well as other causes of secondary HLH, defining further a subpopulation of CD8^+^CD4^dim^ T lymphocytes that produces IFN-γ and correlates with MAS severity (31). While we did not observe these dual-positive cells, our data from scRNA-Seq and mass cytometry now demonstrate that high expression of CD38 and HLA-DR identify cycling CD4^+^ T cells and NK cells as well as CD8^+^ T cells. These cell subsets exhibited strong expression of genes involved in cell proliferation, glycolytic metabolism, and, for CD8^+^ and NK cells, cytotoxicity. Both CD4^+^ and CD8^+^ cycling T cells prominently express *IFNG*. Mirroring the biology of HLH, proliferation and accumulation of these T and NK cell subsets may have a key role in the immune activation and cytokine production in MAS. Additional studies are needed to further specify the relative contribution of these cell subsets to the pathophysiology of MAS.

Notably, CD38^+^HLA-DR^+^ T cells are not unique to HLH and MAS. CD8^+^ T cells that express CD38 and HLA-DR have been described in severe influenza infection, HIV infection, and MIS-C (32, 33, 39). MIS-C is an unique complication of COVID-19 that shares features of MAS, including cytokine storm, cytopenia, and coagulopathy (40–42). Our assessment of pediatric inflammatory conditions demonstrated a greater expansion of CD38^+^HLA-DR^+^ CD4^+^ T cells and CD8^+^ T cells in SD-associated MAS and MAS secondary to other causes than in MIS-C, Kawasaki Disease, and common viral infections. There are also differences within the T cell populations: MIS-C is characterized by a comparable increase in CD38^+^HLA-DR^+^CD4^+^ T cells and their CD8^+^ counterparts, while a predominance of CD8^+^ T cells is observed in MAS. The reason for the difference is unclear, but it is intriguing to speculate that this observation is related to the unique T cell receptor repertoire in MIS-C (43–45).

### A potential synergy of IFN-I and IL-15 in MAS.

The role of IFN-γ is well established in MAS/HLH, in both animal models and human interventional trials. Surrogate markers of IFN-γ activity are used as biomarkers of disease activity, and the monoclonal antibody emapalumab was recently approved for the treatment of HLH (14, 19). In contrast, high levels of IFN-I are typically associated with autoimmune diseases, such as SLE and monogenic interferonopathies (46, 47). Surprisingly, we find that a strong transcriptional signature of IFN-I activity is present in most patients with MAS associated with SD. Since MAS can be triggered by viruses in patients with SD as well as in healthy individuals, we speculate that the production of IFN-I elicited by virus-sensing immune mechanisms may prime the development of pathogenic CD38^+^HLA-DR^+^ T and NK cells in MAS. Supporting this view, IFN-I has been shown to support the expansion of CD4^+^ T cells, CD8^+^ T cells, and NK cells as part of the antiviral response (48–50). Further, IFN-I sustains the immune response by shielding activated T cells from the cytotoxic effects of NK cells (51, 52). Interestingly, IFN-I also primes the production of IL-18 by monocytes after toll-like receptor stimulation, such that IFN-I may further enhance the production of IL-18 in MAS beyond the high levels already seen in SD (53).

Our in vitro studies demonstrated synergistic effects of IFN-I and IL-15 in priming the generation of CD38^+^HLA-DR^+^ T cells and NK cells. Elevated IL-15 levels in the plasma of patients with MAS supports a potential pathogenic role of this cytokine in vivo. IFN-I can induce the expression of IL-15RA by monocytes and dendritic cells (35), which may confer trans presentation of IL-15 to T cells and NK cells. Further studies are needed to delineate the relative contribution of cis presentation and trans presentation of IL-15 in MAS. In contrast, IL-1β, IL-6, IL-18, and IFN-γ did not influence the differentiation of CD38^+^HLA-DR^+^ lymphocytes. Despite the markedly elevated levels of IL-18 in SD and MAS, the capacity of IL-18 to activate T cells and NK cells in SD and MAS may be limited by defective response downstream of the IL-18 receptor (54).

Furthermore, patients with SLE and acute viral infections, which are all conditions associated with high levels of IFN-I, showed higher levels of IL-15 compared with healthy controls. While the degree of IL-15 overproduction is mild compared with MAS, elevated IL-15 levels may be a risk factor for the development of MAS associated with SLE and viral infections. Profiling of MAS associated with other causes in future studies will help us understand whether the proposed synergy of IFN-I and IL-15 is a common feature of MAS.

The concurrent upregulation of IFN-I and IFN-γ may have synergistic effects in propagating the inflammatory response in MAS. IFN-I primes the expression and assembly of essential components of the IFN-γ signaling pathway, including STAT1 (55, 56). Our data illustrate the impact of IFN-I on the generation of CD38^+^HLA-DR^+^ T cell subsets with strong expression of IFN-γ. IFN-γ produced by these activated T lymphocytes may then stimulate antigen presenting cells to establish the cycle of reciprocal immune cell activation that characterizes HLH and MAS. Since the receptor complexes for IFN-I and IFN-γ both signal via the JAK-STAT pathway, simultaneous inhibition of these pathways may help explain the therapeutic effects of JAK inhibitors, which are increasingly used for the treatment of HLH and MAS (37). The cell proliferation and mTORC1 signature of cycling lymphocytes also provide a rationale for the use of mTOR inhibitors such as rapamycin. Our recent studies demonstrated a pathogenic role of mTORC1 signaling in animal models of SD and MAS (38). Beyond their antiproliferative properties, mTOR inhibitors can ameliorate inflammatory diseases by favoring the development and proliferation of regulatory T lymphocytes over effector T lymphocytes through rewiring of cellular metabolism (57). While rapamycin was able to reduce the development of CD38^+^HLA-DR^+^ in vitro, the effects appeared milder than with JAK inhibitors. This may reflect the downstream function of mTOR in lymphocyte proliferation, compared with the upstream role of JAK in the signaling of IFN-I and IL-15.

### Limitations.

Our study encounters limitations of observational human studies, as the ex vivo context does not allow us to intervene directly in the production of IFN-I or in the appearance of cycling lymphocytes in the setting of MAS. The drivers of IFN-I production and aberrant lymphocyte proliferation in MAS were not addressed in our study; future work will be necessary to determine the responsible external and/or internal triggers. While in vitro culture of PBMC with IFN-I and IL-15 can induce a population of T cells with some characteristics of the cycling lymphocytes we observed in patients with MAS, we cannot rule out the involvement of other cytokines or cofactors. Our study does not directly examine the role of T cell receptor activation in MAS, although the combination of IFN-I and IL-15 seems to augment the effects of CD3/CD28 activation. Further, we did not investigate the clonality of CD38^+^HLA-DR^+^ T cell subsets. Clonal expansion has not been associated with MAS, and the study by De Matteis et al. did not observe clonal expansion in the T cell compartment (31). Another limitation of our approach is the relatively small sample size that precludes adjustment for heterogeneity within each group (e.g., medication exposure and disease activity). The analyses and interpretations were also limited to PBMC. Multiomic analysis of affected tissues, such as bone marrow and liver, will be necessary to fully construct the immunologic landscape of MAS.

### Conclusion.

In summary, our study describes a transcriptional signature of IFN-I signaling and an expansion of cycling T lymphocytes and NK cells in MAS associated with SD. We demonstrate that the combination of IFN-I and IL-15 potentiates the generation of CD38^+^HLA-DR^+^ lymphocyte subsets in vitro and provide a mechanistic link between viral infections and MAS in SD that may be targetable by JAK inhibition. These findings together help to elucidate the pathogenesis of MAS in SD.

## Methods

### Study groups and Diagnostic criteria.

The following diagnostic criteria were utilized: 2004 International League of Associations for Rheumatology (ILAR) Classification Criteria for JIA; 2013 Childhood Arthritis and Rheumatology Research Alliance (CARRA) case definition of sJIA; 2016 ACR/EULAR MAS Classification Criteria for MAS complicating sJIA; 1992 Yamaguchi’s diagnostic criteria for AOSD (58); 2017 American Heart Association Guidelines for Kawasaki Disease (59); 1997 American College of Rheumatology (ACR) revised criteria for SLE (60, 61); and the 2020 CDC definition of MIS-C associated with COVID-19 (62). The diagnosis of JDM was determined by presence of a typical skin rash, symmetric muscle weakness, muscle enzyme elevation, and MRI results compatible with diffuse muscle inflammation. Patients in the viral illness group tested positive for at least 1 virus by antigen testing or by PCR on the day of sample collection.

Demographics of healthy controls and patient groups are provided in Supplemental Table 2. Demographic variables were defined by the participants. In the MAS group, 2 patients were diagnosed with MAS associated with AOSD, given disease onset between 16 and 19 years of age. The medical record of each case was reviewed by a team of pediatric rheumatologists. Patients with SD were further stratified into inactive SD, active SD without MAS, or active SD with MAS. Active disease was determined by the treating provider based on findings of arthritis, fever, other features of systemic inflammation, and/or elevated markers of inflammation in laboratory studies.

### Sample collection.

Whole blood was collected in tubes containing K-EDTA (BD Biosciences). Flow cytometry studies were performed using whole blood within 24 hours of sample collection. PBMCs were isolated using Ficoll-Paque (BioLegend) gradient within 18 hours of blood draw. For bulk RNA-Seq, 5 × 10^5^ cells were suspended in RLT buffer (QIAGEN) prior to cryopreservation. The remaining cells were cryopreserved in liquid nitrogen and thawed immediately prior to preparation for scRNA-Seq and mass cytometry.

### Flow cytometry.

Antibodies and sources are listed in Supplemental Table 3. Fresh whole blood (50 μL) was incubated with TruStain FcX (BioLegend) for 5 minutes and stained with an optimized amount of primary antibody or the appropriate isotype control for 15 minutes at room temperature. Samples were resuspended in red blood cell lysis buffer (BioLegend) (4 mL) for 5 minutes, centrifuged, and resuspended in PBS supplemented with 0.1% BSA. Intracellular staining was performed after surface staining using fixation and permeabilization buffer (BioLegend). Antibodies were added to cells after the permeabilization step and samples were incubated for at least 1 hour before analysis. Samples were acquired using a Becton-Dickinson FACS Canto II flow cytometer and analyzed with FCS Express 5 software (De Novo Software). Intact cells were identified by size and singlets were gated for analysis. Isotype control antibodies were used to establish gates to determine cell populations and quantify mean fluorescence intensity.

### scRNA-Seq.

Individuals who participated in scRNA-Seq included: people in the healthy control group (*n* = 5), patients with SD without MAS (*n* = 10; 8 with active disease also analyzed by bulk RNA-Seq, 2 with inactive disease), and patients with SD with MAS (*n* = 9; all 9 were analyzed by bulk RNA-Seq). The 24 samples were processed in 8 scRNA-Seq runs and each run contained samples from at least 2 of the 3 groups. 10× scRNA-Seq (3′) was performed with assistance from the Brigham and Women’s Hospital Center for Cellular Profiling. Cryopreserved PBMCs were thawed and immediately incubated with TruStain FcX (BioLegend) for 10 minutes at 4°C followed by incubation with Zombie viability dye (Invitrogen) and hashing antibodies (TotalseqB Hashtag-1, 2 and 3; Biolegend) for 30 minutes at 4°C. The cells were washed and resuspended in Cell Staining Buffer (BioLegend) for sorting by FACSAriaTM Fusion Cell Sorter (BD Biosciences). Viable single cells were sorted into collection tubes containing PBS (Corning) with BSA (Thermo Fisher Scientific).

Sorted cells were loaded onto a Chromium chip G (10× Genomics) followed by encapsulation in lipid droplets (Single Cell Next GEM kit V3.1, 10X Genomics). The scRNA-Seq libraries were generated according to the manufacturer’s protocol. Using Illumina Novaseq, a single-cell mRNA library was sequenced to an average of 30,000 reads per cell, and HTO (Cell Hashing antibodies) library was sequenced to an average of 5,000 reads per cell. Cell Ranger toolkit (version 3.1.0) was applied to demultiplexing, FASTQ file generation, alignment (using GRCh38 human reference genome), and barcode processing. Further quality control, processing, correction of batch effects, and analysis for the gene matrices were performed by Seurat package (version 4.0.1) (63), Harmony (version 0.1.0) (64), and ggplots package (version 3.3.3) in R (version 4.0.4). Cell-cell communication analysis was performed using CellChat package (version 1.5.0) (29).

### Bulk RNA-Seq.

Individuals who participated in bulk RNA-Seq included: people in the healthy control group (*n* = 18; 10 children, 8 adult), patients with active SD without MAS (*n* = 10), and patients with SD with MAS (*n* = 10). The Smartseq2 platform for low-input RNA-Seq was chosen due to the limited number of cells from patients with MAS. Total RNA was extracted from samples cryopreserved in RLT buffer using Qiagen RNeasy Micro kit (Qiagen). Smart-Seq2 transcriptome libraries were prepared and sequenced by the Broad Institute Genomics Services. Raw sequencing reads were obtained as fastq.gz files. The quality of raw reads was checked by FastQC (v0.11.9). HISAT2 (v2.2.1) was used to align raw reads to the human reference genome (GRch38) from Genome Reference Consortium. Resulting BAM files were sorted and indexed using Samtools (v1.12). The number of reads was mapped to annotated Ensembl genes, and the count was merged to a matrix with HTSeq. The difference between samples was normalized by DESeq(v1.32.0). fGSEA package (v1.18.0) was used to determine differential gene expression and enrichment of pathways according to the hallmark gene set collection. Graphs were generated using ggplot2 (v.3.3.5).

### Mass cytometry.

Individuals who participated in mass cytometry analysis included: people in the healthy control group (*n* = 5), patients with active SD without MAS (*n* = 7), and patients with SD with MAS (*n* = 5). Mass cytometry was performed with assistance from the Longwood Medical Area CyTOF Core; the panel of metal-conjugated antibodies is listed in Supplemental Table 4. All samples were processed and analyzed in a single batch. Thawed cells were washed with Maxpar staining buffer (Fluidigm), incubated with cisplatin live/dead stain (Fluidigm) for 2 minutes, and blocked with anti–human CD16/32 (Biolegend; 163403) before incubation with surface staining antibody cocktail for 30 minutes. After washing with staining buffer, cells were fixed and permeabilized using eBioscience Fixation/Permeabilization kit (Thermo Fisher Scientific), then barcoded using a palladium-based barcode reagent as previously described (65). After washing, samples were pooled and stained with intracellular antibodies. Events were acquired using a Helios mass cytometer (Fluidigm). Data normalization and deconvolution of barcoded staining data was conducted using the normalizer and the single-cell-debarcoder software as described previously (65, 66). OMIQ software was used for data analysis and graphic display.

### Cytokine quantification.

Plasma IL-18 and CXCL9 were measured by ELISA as previously described (15). Plasma IL-15 was quantified by proximity extension assay through Olink. Bioactivity of IFN-I in human plasma was measured using a luciferase reporter cell line as described (67). HEK 293T cells with luciferase expression under the control of IFN-sensitive response element were maintained in DMEM supplemented with penicillin / streptomycin and 10% FBS. Cells (1 × 10^5^ per well) were plated in a 96-well plate and cultured in 200 μL of complete medium with 10% human plasma (from healthy controls or patients) for 18 hours. Duplicate wells were tested for each plasma sample. Cell lysate preparation and quantification of luciferase activity were performed using the BrightGlo reagent (Promega) according to manufacturer’s instructions.

### In vitro stimulation.

Human IL-1β, IL-4, IL-6, IL-12, IL-15, IL-18, IL-27, TNF, IFN-α2, and IFN-γ were purchased from Biolegend. Freshly isolated PBMCs were resuspended in complete RPMI supplemented with penicillin / streptomycin and 10% FBS. Cytokines were added individually and in combination after 1 hour. In some experiments, ruxolitinib (100 nM), tofacitinib (100 nM), rapamycin (1 μM), or vehicle control (DMSO) was added to cells 30 minutes before cytokine stimulation. Cells were analyzed by flow cytometry after 48 hours unless indicated otherwise. For detection of CD107a externalization, PBMCs (2 × 10^5^ per well in 200 μL of complete RPMI in 96-well plate) were stimulated with 1 μL of anti-CD3/CD28 Dynabeads (Thermo Fisher Scientific) in the presence of PE-anti-CD107a antibody (Biolegend) for 3 hours prior to staining with additional surface antibodies and flow cytometry analysis. For detection of Ki-67, PBMC were cultured with IFN-α2 and IL-15, and/or anti-CD3/CD28 Dynabeads for 6 days.

### Statistics.

For quantitative variables, unless indicated otherwise, differences between 2 groups were analyzed by Mann-Whitney U test and for multiple groups by Kruskal-Wallis test. Median and interquartile range are displayed unless noted otherwise. For correction of multiple testing, Dunn’s correction was applied for post-hoc comparisons between groups. All tests were 2-sided, and *P* < 0.05 was considered significant. Statistical analyses were performed using Prism 9.0 software.

### Study approval.

All human subject research was approved by the IRB of Boston Children’s Hospital and Brigham and Women’s Hospital. Informed consent was provided by participants or legal guardians and assent was obtained from patients when appropriate.

### Data availability.

Raw data are available in the Supporting Data Values file. Bulk RNA-Seq and scRNA-Seq data have been deposited in NCBI database of Genotypes and Phenotypes (dbGaP) under accession number phs003310.v1.p1.

## Supplementary Material

Supplemental data

ICMJE disclosure forms

Supporting data values

## Version 1 09/26/2023

In-Press Preview

## Version 2 11/15/2023

Electronic publication
