# Supplementary material for: Type I interferon signature and cycling lymphocytes in macrophage activation syndrome
Source: J Clin Invest. 2023 Nov 15;133(22):e165616. doi: 10.1172/JCI165616 (PMC10645381; doi:10.1172/JCI165616)
Supplement: Supplemental data [file jci-133-165616-s025.pdf]

Type I interferon signature and cycling lymphocytes in macrophage activation syndrome

Huang Z, Brodeur K, Chen L, Du Y et al.

Supplemental Materials

Supplemental Table 1 – 4

Supplemental Figures 1 – 8

Supplemental Table 1. Demographics, laboratory data and medication usage in patients with SD with or without MAS.

	SD without MAS**	SD with MAS***
Demographics		
Total # of subjects	10	10
Age (year)	5.0 ± 4.1	8.7 ± 7.6
Female	50%	50%
Active disease	100%	100%
Laboratory studies*		
WBC (x10^3/mL)	11.3 [8.2, 23.5]	8.9 [4.9, 12.6]
Hemoglobin (g/dL)	10.7 [10.3, 11.1]	9.5 [8.3, 11.2]
Platelet (x10^3/mL)	389 [346, 624]	205 [163, 330]
CRP (mg/dL)	4.6 [0.5, 12.9]	5.8 [1.5, 12.1]
Ferritin (ng/mL)	416 [86, 1066]	2894 [2221, 5906]
AST (U/L)	31 [21, 35]	58 [50, 110]
Triglyceride (mg/dL)	135 [106, 160]	220 [188, 289]
Fibrinogen (mg/dL)	693 [274, 750]	340 [203, 406]
LDH (U/L)	312 [254, 483]	652 [577, 774]
IL-18 (pg/mL)	17220 [7488, 36987]	101546 [71741, 200195]
CXCL9 (pg/mL)	366 [330, 578]	3889 [1501, 10053]
Medications		
Anakinra	20%	30%
Canakinumab	0%	0%
Tocilizumab	0%	0%
Tofacitinib	0%	10%
Corticosteroids	30%	30%
Methotrexate	10%	10%

* Values represent median and interquartile range

** All 10 patients in the active SD without MAS group were analyzed by bulk RNA-seq; 8/10 patients were analyzed by single cell RNA-seq. Two patients with inactive SD were also incorporated in SD without MAS group for single cell RNA-seq analysis.

*** All 10 patients in the MAS group were analyzed by bulk RNA-seq; 9/10 were analyzed by single cell RNA-seq.

Supplemental Table 2. Demographics of healthy controls and patient groups for flow cytometry studies.

		n	Age (year)	Female	Active disease
Healthy Controls	Children	11	9.5 ± 6.3	45%	-
	Adults	9	32.3 ± 6.6	67%	-
inactive sJIA		11	9.8 ± 6.4	55%	0%
active sJIA		11	11.1 ± 5.3	55%	100%
non-sJIA [a]		18	12.1 ± 6.2	72%	72%
MAS		10	9.7 ± 7.9	50%	100%
Other MAS [b]		5	7.4 ± 7.6	40%	100%
SLE		9	14.7 ± 2.2	78%	78%
JDM		11	12.2 ± 5.3	73%	66%
MIS-C [c]		20	8.2 ± 3.3	60%	100%
KD [d]		10	2.5 ± 1.6	50%	100%
Viral infection [e]		18	2.2 ± 2.6	44%	100%

a. The non-systemic juvenile idiopathic arthritis (JIA) group includes patients with oligoarticular JIA, polyarticular JIA and psoriatic arthritis and enthesitis-related arthritis.
b. The other MAS group includes 3 patients with infection-associated MAS, 1 patient with MAS secondary to a described gain-of-function CDC42 variant, and 2 patients with MAS of unclear etiology.
c. Samples for patients with MIS-C were collected before any immunosuppressive therapy (55%), within 48 hours of treatment initiation (35%), or > 48 hours after treatment initiation (10%).
d. The Kawasaki disease group includes 7 patients with complete KD and 3 patients with incomplete KD. One patient had clinical features of Kawasaki shock syndrome.
e. The viral infection group were confirmed to have SARS-COV-2 (n=6), rhinovirus (n=6), adenovirus (n=5), parainfluenza virus (n=5), respiratory syncytial virus (n=1), or human metapneumovirus (n=1). Six individuals tested positive for 2 viruses.

Supplemental Table 3. List of flow cytometry antibodies and sources.

Target antigen	Clone	Conjugate	Cat no.	Lot no.	Source	Dilution
CD3	OKT3	PE	317308	B349598	Biolegend	1:400
CD4	OKT4	FITC	317408	B325364	Biolegend	1:400
CD8	SK1	PE-Cy7	344750	B337146	Biolegend	1:800
CD56	5.1H11	Pacific Blue	362520	B323034	Biolegend	1:200
CD38	HB-7	Brilliant Violet 510	356612	B346260	Biolegend	1:200
HLA-DR	L243	APC	307610	B278650	Biolegend	1:200
CD107a	H4A3	PE	328607	B283847	Biolegend	1:200
Perforin	B-D48	FITC	353309	B352897	Biolegend	1:200
Granzyme A	CB9	Pacific Blue	507208	B268999	Biolegend	1:200
Granzyme B	QA16A02	FITC	372205	B274305	Biolegend	1:200
CD122	TU27	APC	339007	B280345	Biolegend	1:200
CD132	QA17A55	PE	314603	B328365	Biolegend	1:200
CD215	JM7A4	APC	330209	B343828	Biolegend	1:200
Ki-67	Ki-67	Pacific Blue	350511	B279318	Biolegend	1:200
TotalSeq B0251 Hashtag 1	LNH-94; 2M2	RNAseq Hashtag	394631	394631	Biolegend	1 µg per $2X10^6$ cells
TotalSeq B0252 Hashtag 2	LNH-94; 2M2	RNAseq Hashtag	394633	394633	Biolegend	1 µg per $2X10^6$ cells
TotalSeq - B0253 Hashtag 3	LNH-94; 2M2	RNAseqHashtag	394635	394635	Biolegend	1 µg per $2X10^6$ cells

Supplemental Table 4. List of metal isotope-conjugated antibodies for mass cytometry.[+]

Marker	Clone	Isotope	Marker	Clone	Isotope
CD45	HI30	89Y	CD39*	A1	158Gd
CD172ab	SE5A5	111Cd	FPR1	350418	160Gd
CD8a*	RPA T8	112Cd	CD303	REA693	161Dy
CD20*	2H7	113Cd	MARCO	Polyclonal	162Dy
CD4*	RPA T4	114Cd	CCR2	K036C2	163Dy
CD3*	UCHT1	115In	CD141	M80	164Dy
CD56*	NCAM16.2	116Cd	CD38*	HIT2	165Ho
Siglec-6	767329	141Pr	CLEC9A	REA976	166Er
TLR4	610015	142Nd	CD84	CD84.1.21	167Er
CD36	5-271	143Nd	HO-1	HO-1-1	168Er
CD64	10.1	144Nd	CX3CR1*	REA385	169Tm
CD163	REA812	145Nd	PD-L1	29E.2A3	170Er
CD74	LN2	146Nd	CD206	19.2	171Yb
CD86	IT2.2	147Sm	CD170	1A5	173Yb
CD1c	L161	148Nd	Ki67*	8D5	174Yb
CD1d	51.1	149Sm	CD85i	586326	175Lu
CD11c	Bu15	150Nd	CD68	Y1/82A	176Yb
CD123	6H6	151Eu	CD180	MHR73-11	194Pt
CD14*	M5E2	152Sm	HLA-DR*	L243	195Pt
CD85d	42D1	153Eu	FOLR2	94b/FOLR2	196Pt
Siglec-1	7-239	155Gd	CD115	61708	198Pt
XCR1	1097A	156Gd	CD11b*	ICRF44	209Bi
CD16*	3G8	157Gd			

[+] all antibodies were prepared by the Longwood Medical Area CytoF Antibody Resource Core

* markers included in the UMAP analysis of T cell and NK cell subsets

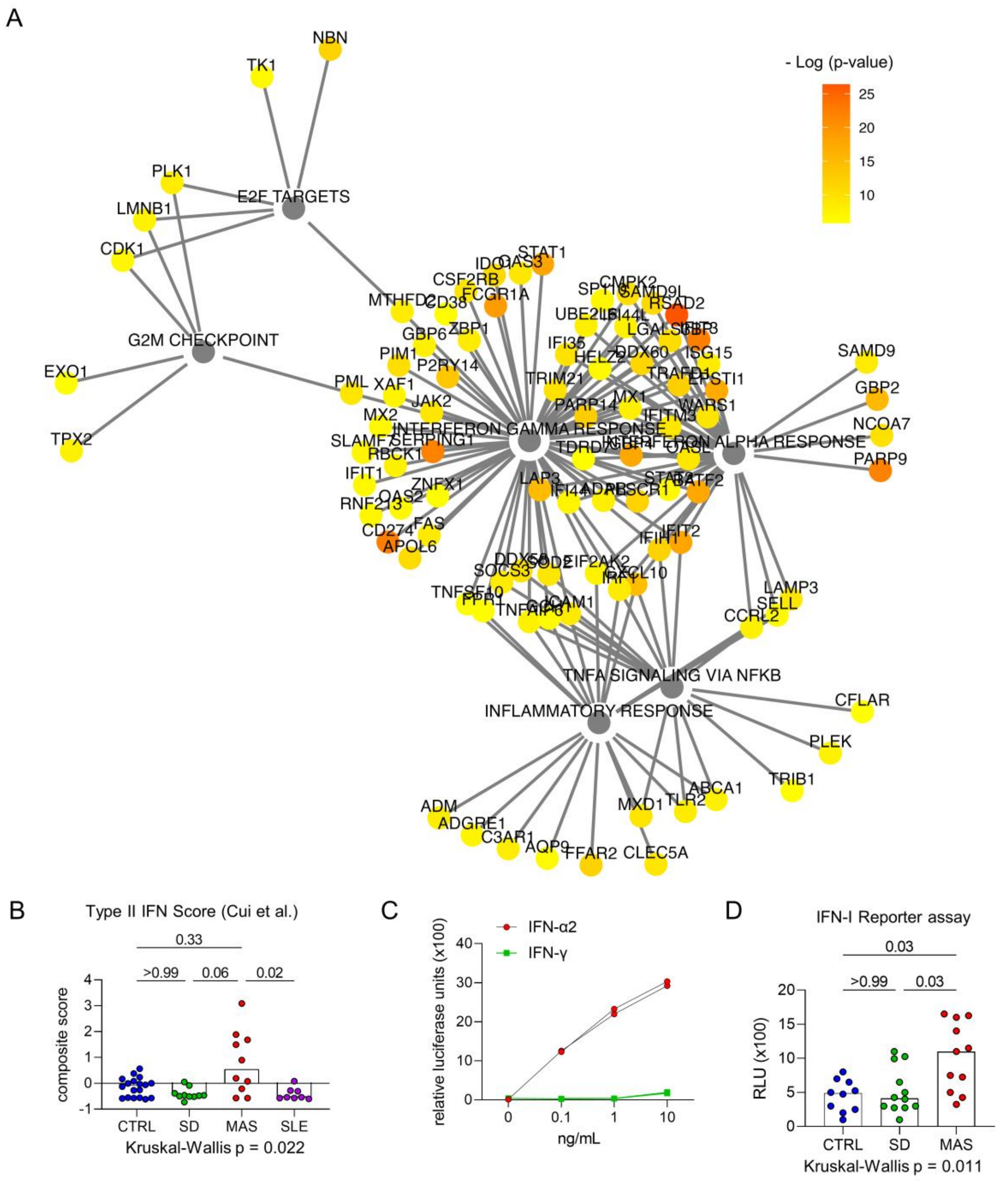
A
- Log (p-value)
25
20
15
10
E2F TARGETS
G2M CHECKPOINT
INTERFERON GAMMA RESPONSE
TNFA SIGNALING VIA NFKB
INFLAMMATORY RESPONSE
NBN
TK1
PLK1
LMNB1
CDK1
EXO1
TPX2
MTHFD2
CSF2RB
IDO1
STAT1
FCGR1A
CD38
GBP6
ZBP1
PIM1
P2RY14
PML
XAF1
JAK2
MX2
SLAMF7
SERPING1
RBCK1
IFIT1
ZNFX1
OAS2
RNF213
FAS
CD274
APOL6
SAMD9
GBP2
NCOA7
PARP9
IFI35
ISG15
TRIM21
PARP14
MX1
IFITM3
WARS1
TDRD7
LAP3
IFI44
IFIT2
SOCS3
TNFSF10
LAMP3
SELL
CCRL2
CFLAR
PLEK
TRIB1
ABCA1
TLR2
MXD1
ADM
ADGRE1
C3AR1
AQP9
FFAR2
CLEC5A
B
Type II IFN Score (Cui et al.)
0.33
>0.99
0.06
0.02
composite score
4
3
2
1
0
-1
CTRL
SD
MAS
SLE
Kruskal-Wallis p = 0.022
C
IFN-α2
IFN-γ
relative luciferase units (x100)
40
30
20
10
0
0
0.1
1
10
ng/mL
D
IFN-I Reporter assay
0.03
>0.99
0.03
RLU (x100)
20
15
10
5
0
CTRL
SD
MAS
Kruskal-Wallis p = 0.011

Supplemental Figure 1. Presence of IFN-I and IFN-γ gene signatures in PBMC from patients with SD-associated MAS. A) Cytoscape network analysis of the top 200 genes enriched in the MAS group (n=10) compared to healthy controls (n=18) using the Hallmark gene set collection. B) Comparison of composite gene set score derived from a published IFN-γ signature gene set. C) Quantification of luciferase activity in 293T IFN-I reporter cells stimulated with the indicated concentrations of IFN-α2 or IFN-γ (n = 2 for each concentration). D) Quantification of luciferase activity in 293T-ISRE-luciferase reporter cells cultured with 20% plasma from healthy controls (n=10) and patients with SD with (n=11) or without MAS (n=17). Statistical analysis: Bars in panels B and D represent the median. Kruskal-Wallis test was used for comparison of multiple groups and Dunn's correction was applied for the indicated comparisons.

Supplemental Figure 2

A

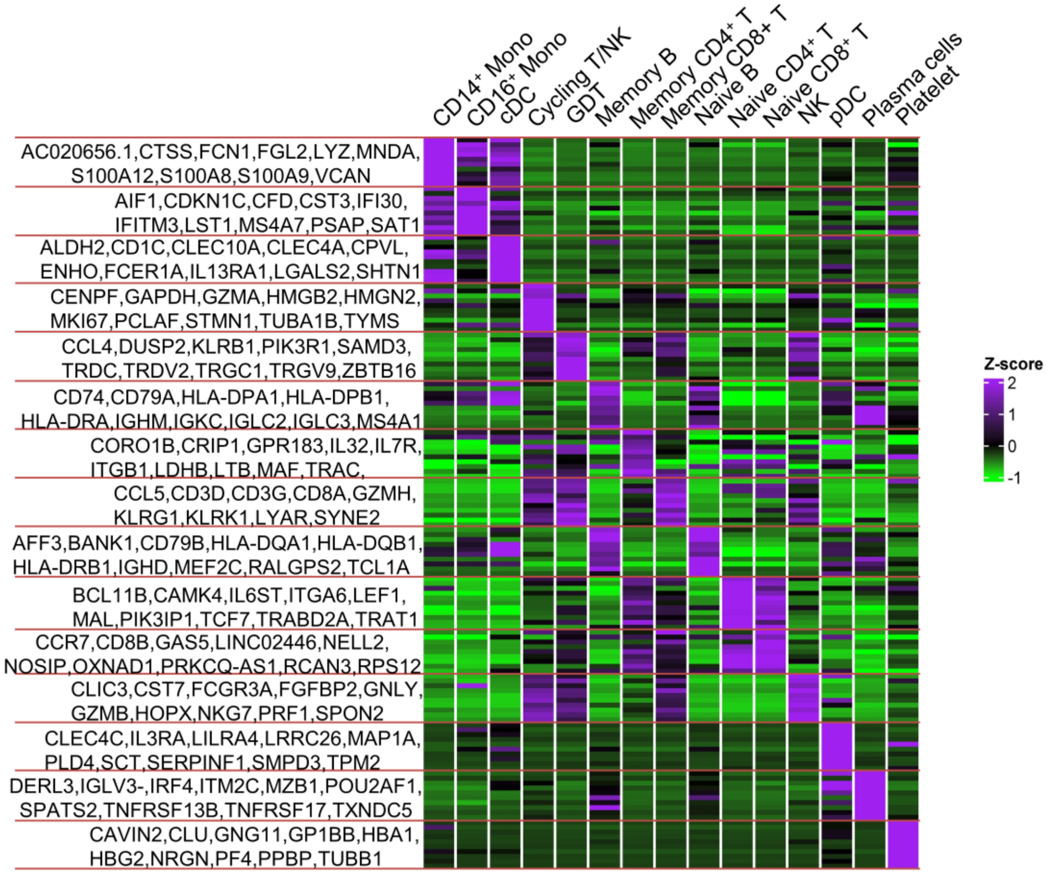

B

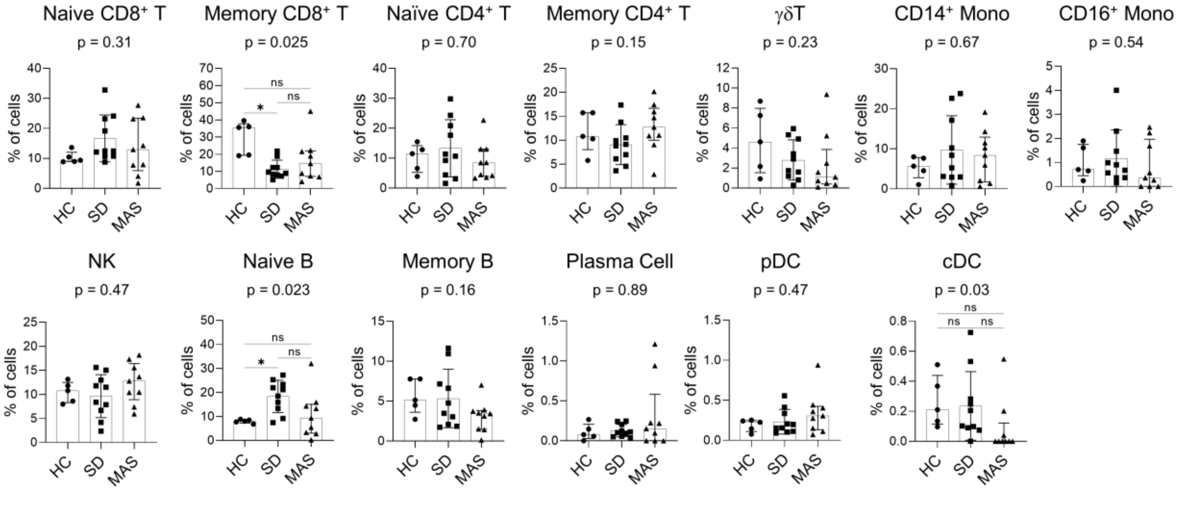

Supplemental Figure 2. scRNA-seq of PBMC from healthy controls and patients with SD with or without MAS. A) Heatmap display of top 10 differentially expressed genes unique to each cell subset. B) Quantification and comparison of cell subsets in healthy controls (n=5) and patients with SD without MAS (n=10) or with MAS (n=9). Statistical analysis: Bars represent the median and error bars indicate interquartile range. Kruskal-Wallis test was used for comparison of multiple groups and Dunn's correction was applied for the indicated comparisons.

Supplemental Figure 3.

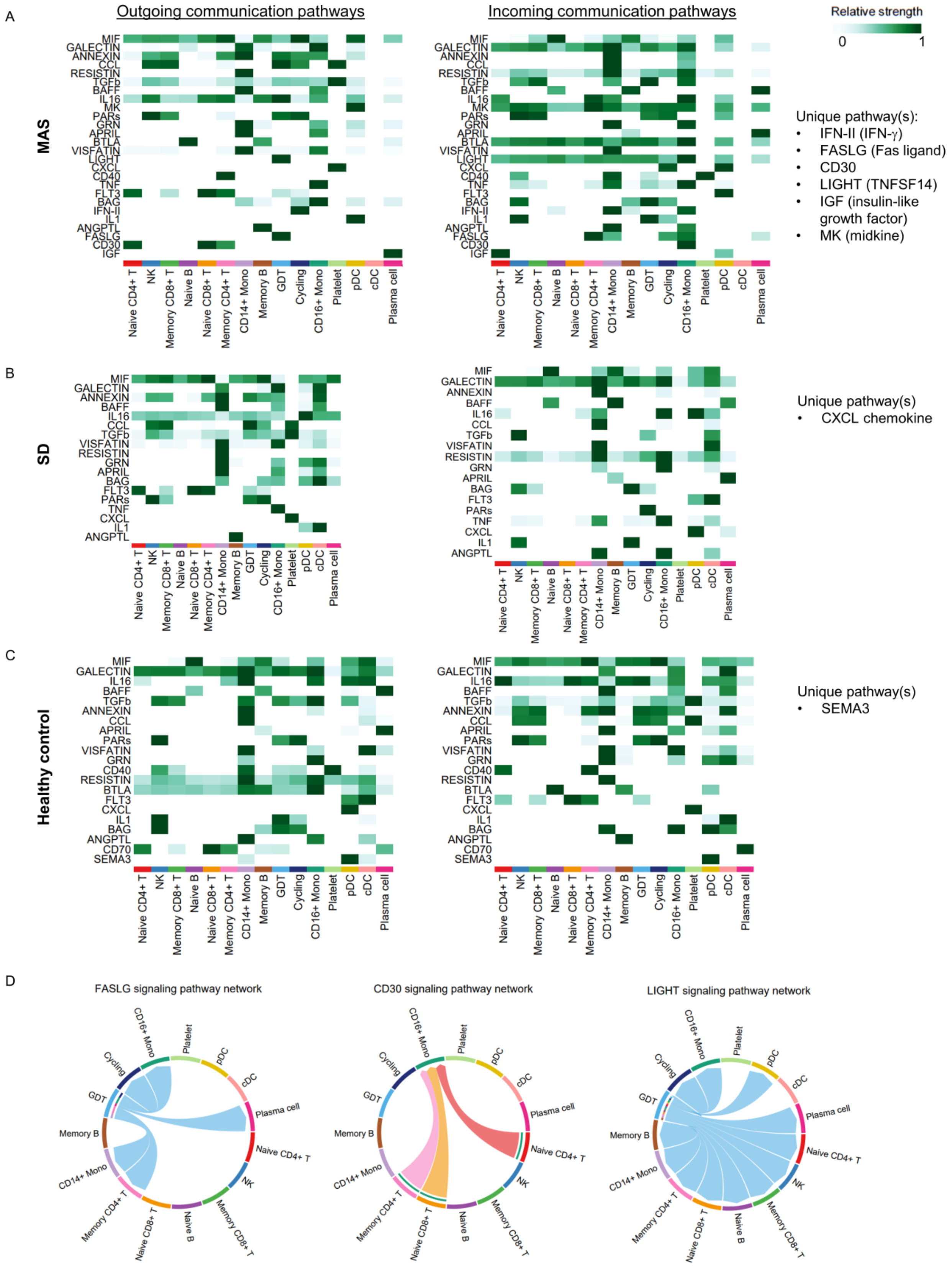

Supplemental Figure 3. Analysis of cell-cell interactions from scRNA-seq of SD and MAS. A-C) heatmap display of outgoing and incoming signal pathways in PBMC cell subsets based on CellChat analysis of scRNA-seq data from healthy controls (panel A), patients with active SD without MAS (panel B), and active SD with MAS (panel C). Pathway(s) unique to each group are displayed on the right. D) Chord diagrams illustrating the Fas ligand, CD30 and LIGHT pathway interactions in PBMC subsets from patients with MAS.

Supplemental Figure 4.

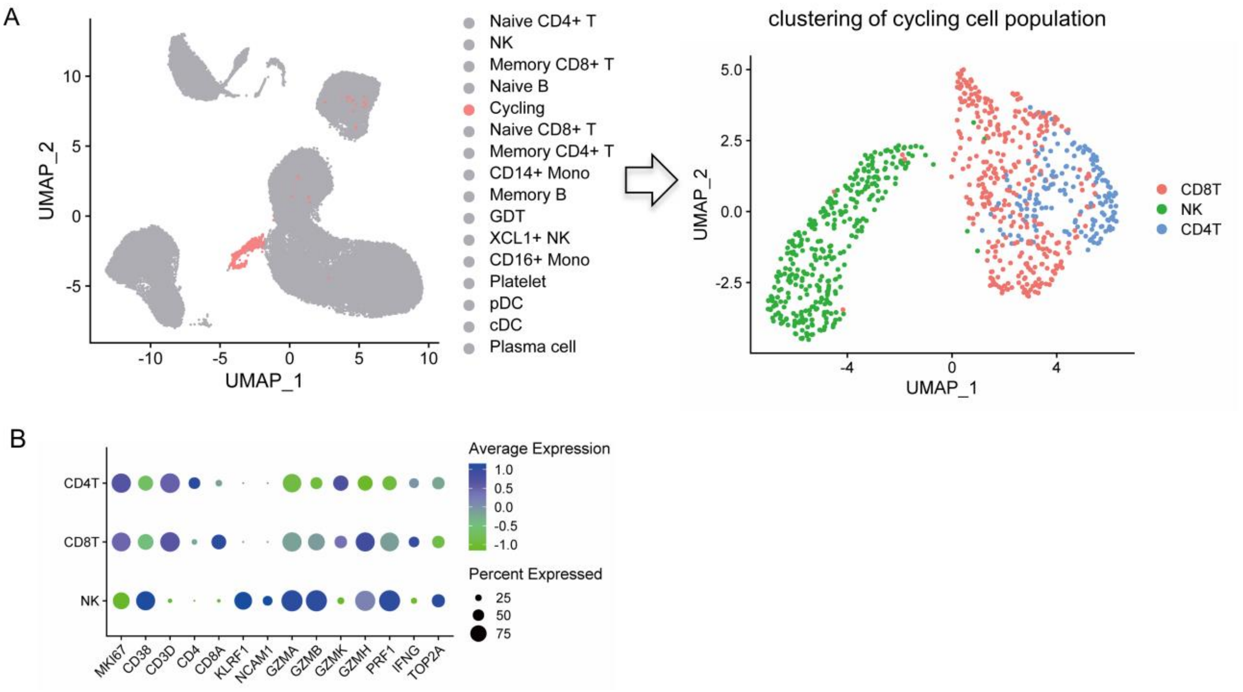

Supplemental Figure 4. scRNA-seq of cycling lymphocyte subsets. A) UMAP display of cell subsets within the cycling lymphocyte population. B) Cluster plot of lineage-defining markers for cycling CD4$^+$ T cell, CD8$^+$ T cell and NK cell subsets.

Supplemental Figure 5.

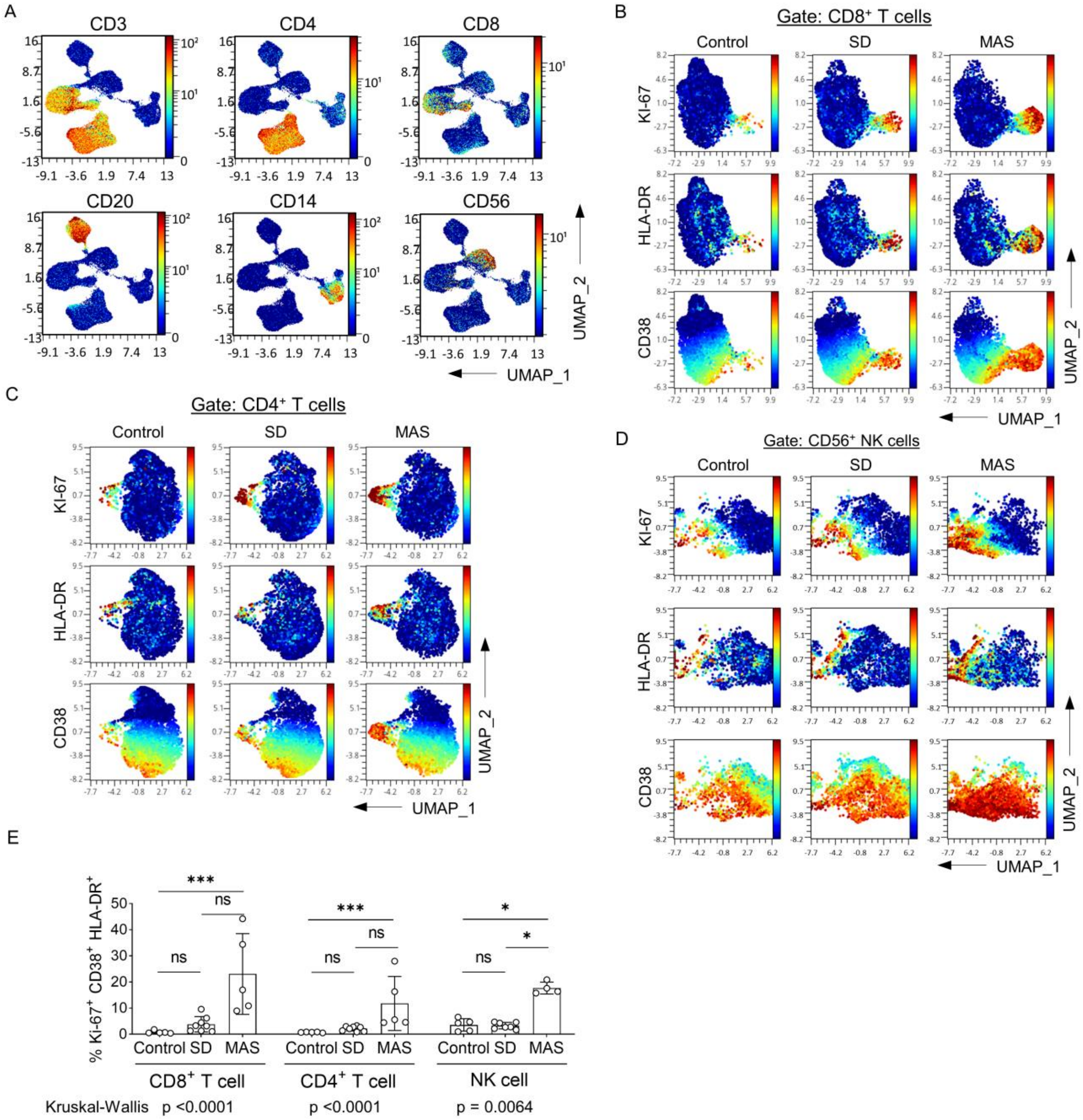

Supplemental Figure 5. Identification of $CD38^{+}HLA\text{-}DR^{+}$ cycling lymphocytes by mass cytometry. A) UMAP display of PBMC concatenated from healthy controls (n=5), patients with SD without MAS (n=7) and with MAS (n=4). Color denotes the expression of the indicated lineage markers. UMAP display of B) $CD8^{+}$ T cells, C) $CD4^{+}$ T cells, and D) NK cells comparing PBMC concatenated from healthy controls and patient groups. Color denotes the expression of indicated markers including MKI67, CD38 and HLA-DR. E) Quantification of $MKI67^{+}CD38+HLA\text{-}DR^{+}$ cells within T cell and NK cell populations and comparison among healthy controls, patients with SD without MAS, and patients with MAS. Statistical analysis: Bars represent the median and error bars indicate interquartile range in panel E. Kruskal-Wallis test was used for comparison of multiple groups and Dunn's correction was applied for the indicated comparisons.

Supplemental Figure 6.

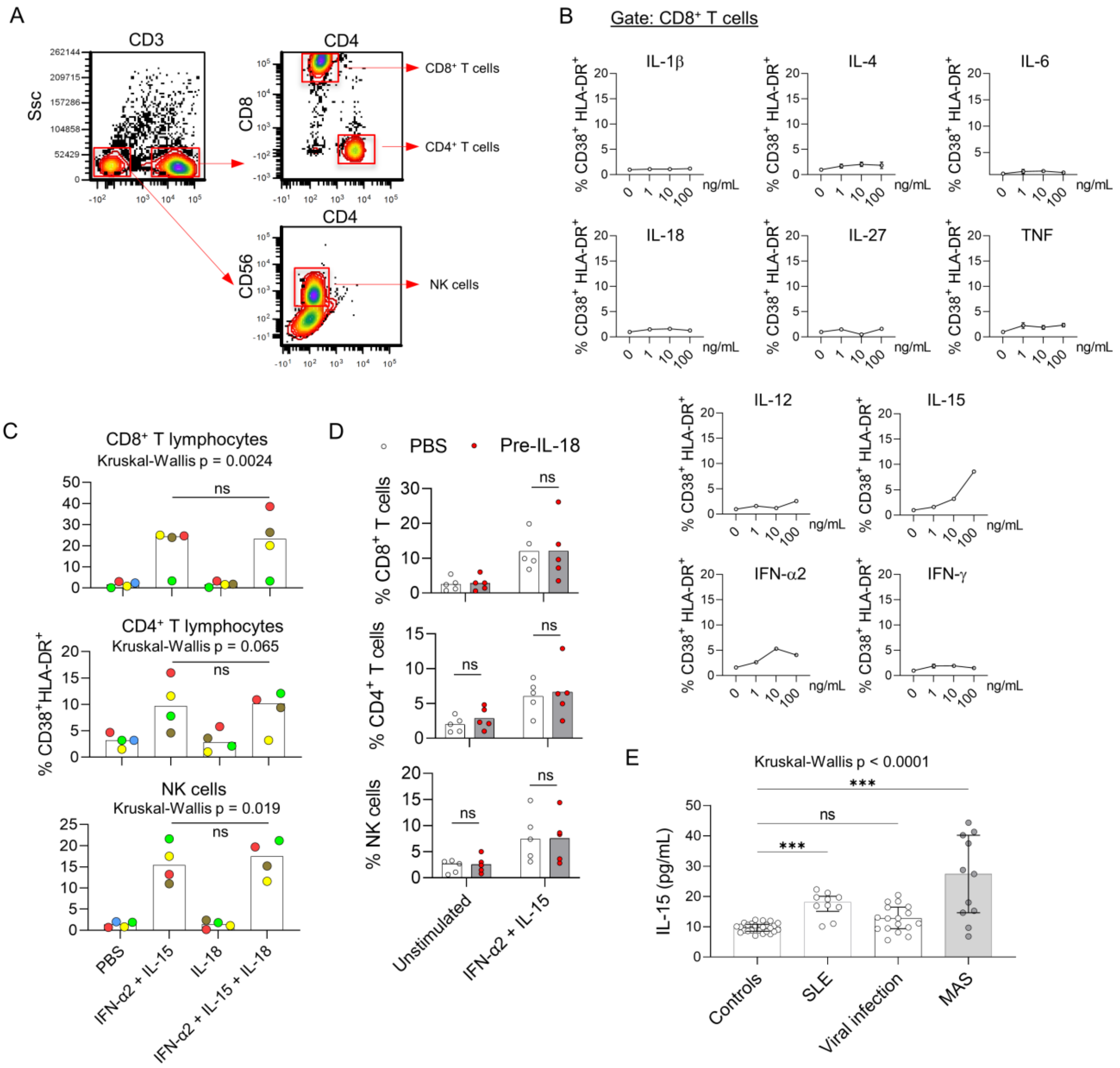

Supplemental Figure 6. Analysis of $CD38^{+}HLA\text{-}DR^{+}$ lymphocytes and induction by cytokines. A) Gating strategy to identify $CD4^{+}$ T cells, $CD8^{+}$ T cells and NK cells in PBMCs. B) Induction of $CD38^{+}HLA\text{-}DR^{+}CD8^{+}$ T cells by coculturing PBMC from a healthy donor with various cytokines at the indicated concentrations for 2 days. C) Quantification of $CD38^{+}HLA\text{-}DR^{+}$ lymphocyte subsets induced by coculturing PBMC from healthy controls (n=5) with IL-15 (10 ng/mL), IFN-α2 (10 ng/mL), and/or IL-18 (100 ng/mL) for 2 days. Each color in panel B represents a unique healthy donor (n=4). Results for each donor represent the average of duplicate samples. D) Quantification of $CD38^{+}HLA\text{-}DR^{+}$ lymphocyte subsets induced by coculturing PBMC from healthy controls (n=4-5) with IL-15 (10 ng/mL) and IFN-α2 (10 ng/mL), with or without IL-18 pre-treatment (100 ng/mL; for 24 hours). Cells were analyzed 48 hours after IFN-α2 and IL-15 stimulation. E) Plasma IL-15 levels in healthy controls (n=25), patients with SLE (n=11), acute viral infection (n=17), or active SD with MAS (n=11; previously shown in Figure 6C) as measured by proximity extension assay. Statistical analysis: Bars represent the median and error bars indicate interquartile range in panels C, D and E. Kruskal-Wallis test was used for comparison of multiple groups and Dunn's correction was applied for the indicated comparisons (panels C and E). Mann-Whitney U test was applied for panel D. * $p<0.05$, ** $p<0.01$, *** $p<0.001$.

Supplemental Figure 7.

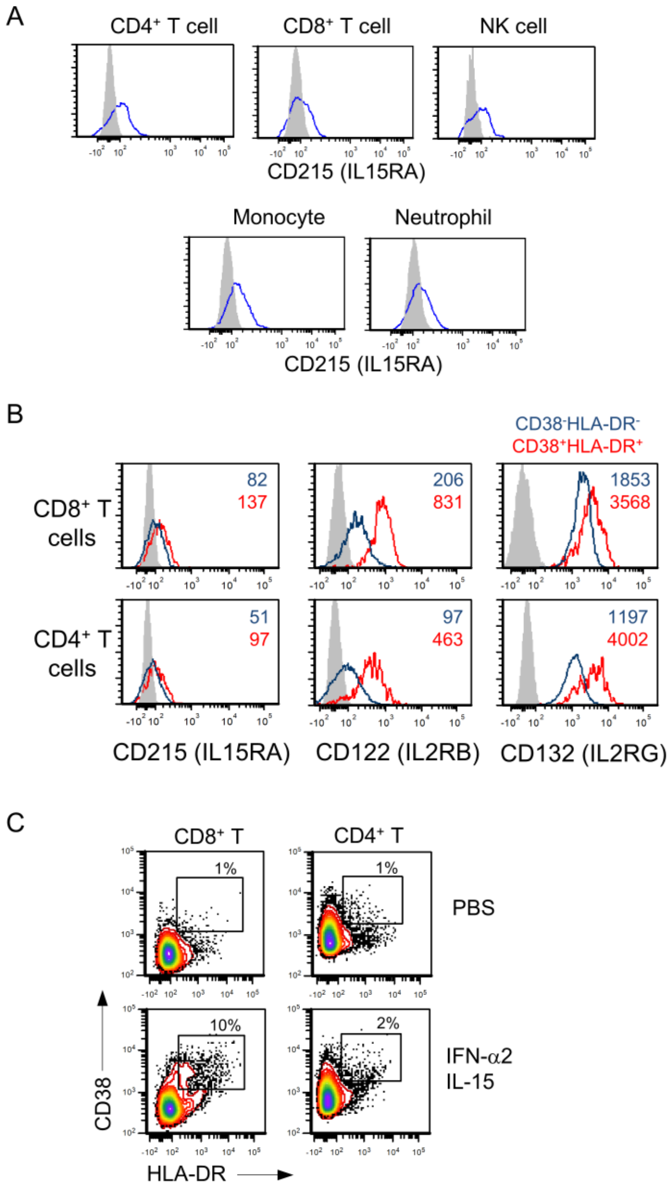

Supplemental Figure 7. Expression of IL-15 receptor components in $CD38^+HLA\text{-}DR^+$ lymphocytes. A) Representative histogram illustrating IL-15RA (CD215), IL2RB (CD122) and IL2RG (CD132) expression by $CD38^+HLA\text{-}DR^+$ and $CD38^-HLA\text{-}DR^-$ T lymphocytes within the $CD8^+$T cell or $CD4^+$ T cell population from a patient with SD-associated MAS. Gray shade represents isotype controls and mean fluorescence intensity is indicated. Results are representative of data from 2 patients with SD-associated MAS. B) Representative histogram illustrating IL-15RA (CD215) expression by PBMC subsets from a healthy donor. Gray shade represents isotype controls. Results are representative of data from 4 healthy controls. C) Representative flow cytometry plot of $CD38^+HLA\text{-}DR^+$ T lymphocyte subsets induced by coculturing purified T lymphocytes from a healthy control with PBS, IL-15 (10 ng/mL), and/or IFN-α2 (10 ng/mL) for 2 days. Results are representative of data from 3 healthy controls.

Supplemental Figure 8.

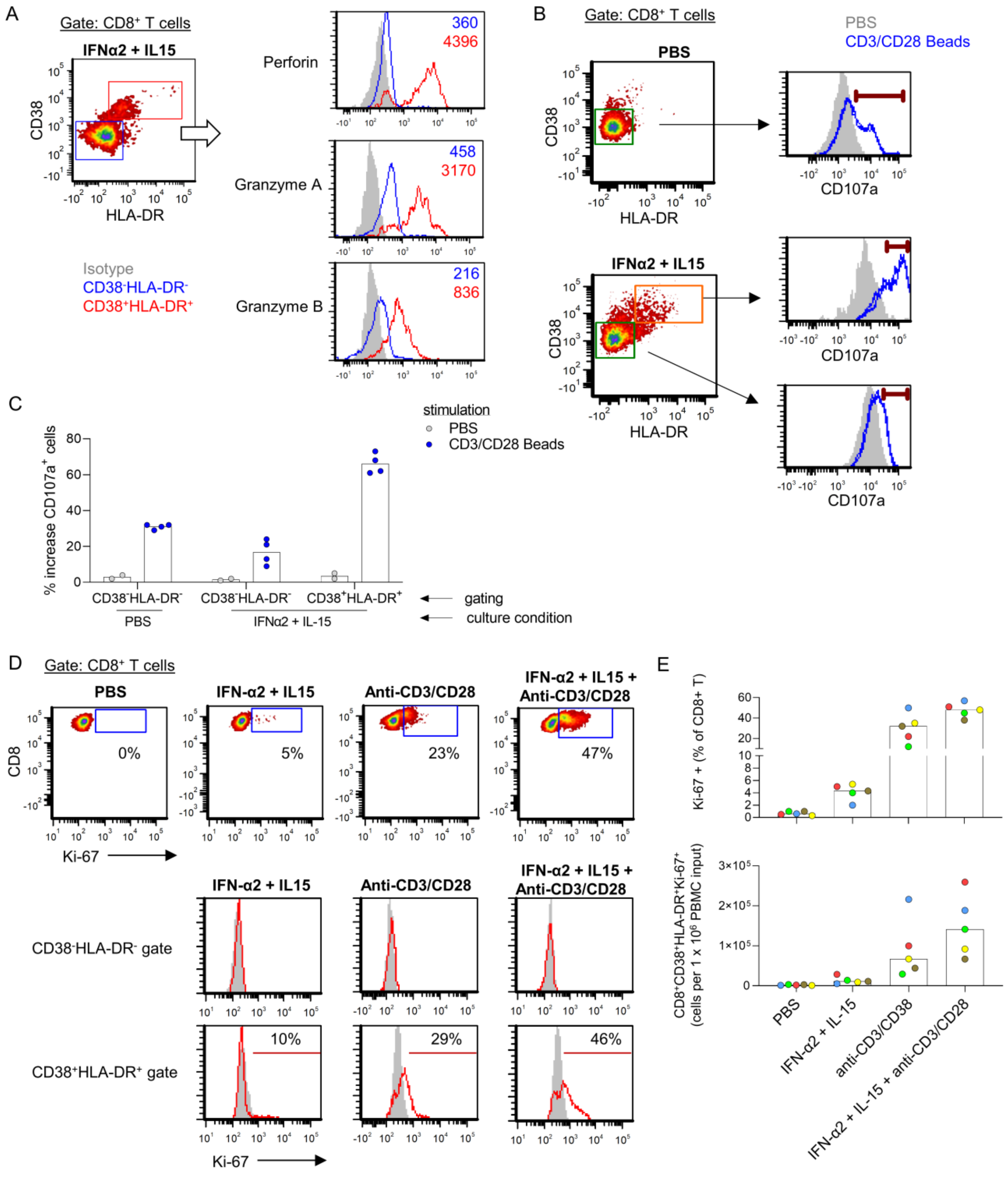

Supplemental Figure 8. Functional characterization of $CD38^+HLA\text{-}DR^+$ lymphocytes. A) Quantification of perforin, granzyme A and granzyme B expression in $CD38^+HLA\text{-}DR^+CD8^+$ (red gate) and $CD38^-HLA\text{-}DR^-CD8^+$ T cells (blue gate) by Intracellular staining. PBMC were cocultured with IFN-α2 + IL-15 for 2 days. Gray shade indicates isotype staining. B) Flow cytometry plots and C) quantification of surface CD107a expression in $CD38^+HLA\text{-}DR^+CD8^+$ and $CD38^-HLA\text{-}DR^-CD8^+$ T cells at baseline (gray shade) and 3 hours after stimulation with CD3/CD28 beads (duplicate samples represented by blue lines). PBMC were cultured with PBS (left panel) or IFN-α2 + IL-15 (right panel) for 2 days. Data in panels A and B are representative of 2-3 independent experiments. Bars in panel C represent the mean. D) Flow cytometry plots of intracellular KI-67 expression in $CD8^+$T cells (upper panel) and comparison of KI-67 in $CD38^+HLA\text{-}DR^+CD8^+$ and $CD38^-HLA\text{-}DR^-CD8^+$ T cells (lower panel) 6 days after stimulation with IFN-α2 + IL-15 and/or anti-CD3/CD28 beads. E) Relative quantification of Ki-67^+ cells (as % of $CD8^+$T cells) and absolute quantification (as number of $CD38^+HLA\text{-}DR^+CD8^+$ Ki-67^+ cells per $1x10^6$ PBMC input) 6 days after stimulation with IFN-α2 + IL-15 and/or anti-CD3/CD28 beads. Each color in panel E represents a unique healthy donor (n = 5). Results for each donor represent the average of duplicate samples.
